# Beeswax-Modified Textiles: Method of Preparation and Assessment of Antimicrobial Properties

**DOI:** 10.3390/polym12020344

**Published:** 2020-02-05

**Authors:** Justyna Szulc, Waldemar Machnowski, Stanisława Kowalska, Anita Jachowicz, Tomasz Ruman, Aleksandra Steglińska, Beata Gutarowska

**Affiliations:** 1 Department of Environmental Biotechnology, Lodz University of Technology, 90-924 Łódź, Poland; jachowicz.anita@gmail.com (A.J.); 190611@edu.p.lodz.pl (A.S.); beata.gutarowska@p.lodz.pl (B.G.); 2 Institute of Material Science of Textiles and Polymer Composites, Lodz University of Technology, Zeromskiego 116 St., 90-924 Łódź, Poland; waldemar.machnowski@p.lodz.pl (W.M.); stanislawa.kowalska@p.lodz.pl (S.K.); 3 Faculty of Chemistry, Rzeszów University of Technology, Powstańców Warszawy Ave. 6, 35-959 Rzeszów, Poland; tomruman@prz.edu.pl

**Keywords:** beeswax, antimicrobial properties, modified textiles, microorganisms

## Abstract

In this work, beeswax was used for the first time for finishing polyester/Cotton/Viscose blend fabric and polyester fabric. The aims of the study were: (1) to characterize the composition of beeswax (using Gas Chromatography Mass Spectrometry, GC-MS and ^109^AgNPET laser desorption/ionization mass spectrometry (LDI MS); (2) to develop a laboratory method for applying beeswax; (3) to assess the antimicrobial activity of beeswax fabrics against bacteria and fungi (AATCC 100–2004 test); and (4) to assess the properties of textiles modified by beeswax. Beeswax was composed of fatty acids, monoacyl esters, glyceride esters and more complex lipids. The bioactivity of modified fabrics was from −0.09 to 1.55. The highest biocidal activity (>1) was obtained for both fabrics against *A. niger* mold. The beeswax modification process neither affected the morphological structure of the fibers (the wax evenly covered the surface of the fibers) nor their color. The only statistically significant changes observed were in the mechanical properties of the fabrics. The results obtained indicate that modification of fabrics with beeswax may endow them with biocidal properties against molds, which has practical applications, for example, for the prevention of skin mycoses in health and social care facilities.

## 1. Introduction

The structure of most textiles (large surface area to volume ratio and the ability to retain moisture), especially those made of natural fibers such as cotton, wool and silk, provide an ideal surface area for microorganism growth. This leads to a range of undesirable effects both on the textile and on users [1]. These effects include unpleasant odors, stains and discoloration, weakness in mechanical strength and an increased likelihood of users getting infected with pathogenic bacteria and fungi [2,3]. These microbial infections are of great concern, especially for textiles used in hospitals and other health care institutions, laboratories, sports clothing, water purification systems and in the food industry [1].

A number of organic and inorganic chemical substances (e.g., quaternary ammonium compounds, polybiguanides, triclosan, metals and metallic salts and chitosan; N-phenyl-male-imide in thiolmaleimide click reaction) with antimicrobial activity are used in textiles. These reduce or completely inhibit the growth of microorganisms [2,4,5,6,7]. Many scientific and technological solutions, such as the inclusion of antimicrobial agents in a textile’s polymeric fibers or grafting them onto the polymer surface, are used for producing textiles with antimicrobial properties [8]. Most often these are added to the fabric during dyeing, finishing or final rinsing of textiles.

Recently, interest in textiles using the phenomenon of photocatalysis has increased.

Ding et al. (2018) prepared visible-light-response Ag/AgCl photocatalyst on cotton fabric using polydopamine (PDA) as adhesive agent and reducing agent through chemical self-polymerization and reduction-oxidation reaction. They showed an outstanding photocatalytic activity of modified cotton (95% of C.I. RB-19 dye degradation rate within 180 min). The authors expected that the modification would have applications in water/air purification with efficient recycling and reuse of the photocatalysts; however, they did not confirm its antimicrobial activity [9].

Ding et al. (2018) synthesized novel hierarchical Z-scheme photocatalyst Ag@AgBr/BiPO_4_/r-GO by in situ deposition of AgBr onto the surface of BiPO_4_/r-GO precursor and followed by photo-reduction of AgBr into Ag@AgBr [10]. Another highly effective photocatalysts (Bi_2_MoO_6_/Ag/AgCl) was prepared by modifying Bi_2_MoO_6_ flower-like microspheres with Ag/AgCl [11]. However, these new solutions in the field of photocatalysis have not yet used to modification of textiles. Also they have not been microbiologically tested.

In the past few years, the desire of consumers for comfort, hygiene and well-being, concerning odor control and pathogen protection, has created a large and fast growing market for antimicrobial textiles. Given growing concerns about microorganism resistance to chemical compounds, increased incidence of allergies and the toxic effects of biocides on the environment, new eco-friendly substances are being sought for endowing textiles with antimicrobial properties [6].

For this purpose so far natural oils such as essential oils and other herbal products have been most often used [12]. High antimicrobial activity against gram-positive and gram-negative bacteria and fungi was proven i. a. for essential oil extracted from *Tithonia diversifolia* (*Asteraceae*) flower [13].

Recently, beekeeping products: honey, propolis, pollen, venom, royal jelly and others have become very popular for many different applications [8].

Beeswax has an extremely wide spectrum of useful applications and occupies a very special place among plant and animal waxes. It is a complex product produced by the species *Apis mellifera* and *Apis cerana* which secretes it in liquid form through their special wax glands [14]. It is nearly white; only after contact with honey and pollen does it assume a variably intense yellowish color and over time turns brown. When it comes in contact with air, it solidifies into scales, which allows the formation of the honeycomb structure. Beeswax is a natural wax composed of a mixture of esters (67 wt%) hydrocarbons (14 wt%), fatty acids (12 wt%), alcohol (1 wt%) and other compounds like aromatic substances and pigments (6 wt%) [15,16,17]. Products from bees have been recognized across the world for their skin-healing properties. They have been used to treat skin disorders, infections, wounds and burns, eczema and inflammation [18,19,20,21,22]. It is worth emphasizing that beeswax is considered a GRAS substance (generally recognized as safe) by the U.S. Food and Drug Administration [23].

The majority of beeswax produced is used for technical purposes (candles, modelling, polishes, etc.). In addition, it is also used in cosmetics, food packaging, processing and preservation (natural food additive E 901) and in medicine (coating pills, for its antimicrobial properties) [24,25]. The antimicrobial activity of beeswax has been documented in European and Asian holistic remedies for centuries. It was found that beeswax is effective against gram-positive and gram-negative bacteria as well as fungi. The antimicrobial effects of beeswax have been found against bacteria from the genera *Bacillus, Escherichia, Listeria, Proteus, Pseudomonas, Salmonella, Staphylococcus*; yeast from the genera *Candida, Rhodotorula*; and molds from the genera *Aspergillus* and *Geotrichum* [25,26].

Based on its documented antimicrobial properties, positive effects on human skin and its natural origin, we modified fabrics for the first time using beeswax, in this study. The aim of this study was (a) to characterize the chemical composition of beeswax, (b) to develop a laboratory method for applying beeswax to fabrics, (c) to assess the antimicrobial activity of beeswax-modified fabrics against selected microorganisms and (e) to analyze the properties (morphological, mechanical and optical properties) of beeswax-modified textiles.

## 2. Materials and Methods

### 2.1. Beeswax

Beeswax came from a private apiary in central Poland in the province of Lodz (year of collection: 2017). It was collected and purified from solid particles by the apiary owner. The melting point of the beeswax used was 61 ± 1 °C.

### 2.2. Analysis of Compounds in Beeswax Using ^109^AgNPET SALDI MS

#### 2.2.1. Materials

Silver-109 (min. 99.75% of ^109^Ag) isotope was purchased from BuyIsotope (Solna, Sweden) and transformed to trifluoroacetate salt using standard methods (this involved dissolving in HNO_3_, followed by ^109^AgOH formation and reaction with trifluoroacetic acid) and recrystallized from the THF/hexane system and 2,5-Dihydroxybenzoic acid (DHB) was purchased from Sigma-Aldrich (Poznan, Poland). Steel targets were machined from stainless steel. The surfaces of the targets were polished using P150 to P2000 grit (ISO/FEPA Grit designation) to produce a mirror-like appearance. All other chemicals were purchased from Sigma-Aldrich (Poznan, Poland) (97–99% purity). All solvents were of HPLC quality, except for water (Ultrapure Water, Merck, Warsaw, Poland) and methanol (LCMS grade, Fluka, Poznan, Poland). The silver-109 nanoparticles were synthesized on the surface of steel targets as described in our publication [27].

#### 2.2.2. Sample Preparation and Handling

Beeswax (50 mg) was suspended in isopropanol (1 mL) and vortexed for 3 min. The suspension was then filtered through syringe filters (0.45 µm pore size) into another Eppendorf vial. A 1 µL solution was then placed on the target plate, air dried and inserted into the MS apparatus.

#### 2.2.3. LDI Mass Spectrometry

LDI-ToF mass spectrometry experiments were performed in reflectron mode using a Bruker Autoflex Speed time-of-flight mass spectrometer (Bruker Autoflex Speed, Bruker Daltonics, Bremen, Germeny) equipped with a SmartBeam II laser (355 nm). Laser impulse energy was approximately 100–150 μJ, laser repetition rate was 1000 Hz. The number of laser shots was 10,000 for each measured spot. Measurement range was *m*/*z* 80–2000. Suppression was turned on typically for ions of *m*/*z* lower than 80. The first accelerating voltage was held at 19 kV and the second ion source voltage at 16.7 kV. Reflector voltages used were 21 kV (the first) and 9.55 kV (the second).

#### 2.2.4. MS Data Handling and Compound Identification

Spectra were calibrated and analyzed with FlexAnalysis (version 3.3) using a centroid calibration model. Mass calibration (enhanced cubic calibration) was performed using internal standards (silver-109 ions and clusters from ^109^Ag^+^ to ^109^Ag_18_^+^). Signals were then checked against LipidMaps database within a 10 ppm window. The list of putatively identified compounds is available in Supplementary Materials.

### 2.3. Modification of Textiles with Beeswax and Their Properties

#### 2.3.1. Textiles

We used two fabrics in this study: a polyester/cotton/viscose blend (Fabric 1) and a polyester fabric (Fabric 2). These types of fabrics were selected because of their suitability for a number of different applications and susceptibility to colonization and destruction by fungi and bacteria. The short description of the tested fabrics is given in Table 1.

#### 2.3.2. Preparation and Application of Beeswax Suspension

Beeswax was suspended in a mixture containing isopropanol and water in a 1:7 ratio using glyceryl stearate (Brenntag, Kędzierzyn-Koźle, Poland) as the dispersing agent. One liter of the suspension was used to impregnate the fabrics contained 30 g of beeswax. Impregnation was performed at room temperature using a horizontal laboratory padding machine. The fabrics were first immersed in the beeswax suspension and then squeezed by passing between the rollers of the padding machine to remove excess liquid. The wet pick-up (the amount of liquid absorbed by a fabric after it has been dipped and padded as a percentage of the weight of the dry fabric) was approximately 100% and 75% for Fabric 1 and Fabric 2, respectively. The impregnated fabric samples were dried at room temperature and then heat-treated in a laboratory dryer at 120 °C for 1 min. The percentage content of beeswax in the fabric samples, after the modification process, was determined based on the concentration of beeswax in the suspension (30 g/L) and the wet pick-up value. The mean final beeswax content in Fabric 1 and Fabric 2 was 3.00% and 2.25%, respectively.

#### 2.3.3. Microscopic Analyzes of Fabrics Modified with Beeswax

We analyzed the distribution of the beeswax in the internal structure of the fabric (i.e., between the warp and weft threads and on the individual fiber surfaces) using a scanning electron microscope, NOVA NANOSEM 230 (FEI, Eindhoven, Netherlands). All fabric samples, both modified and unmodified with beeswax, were coated with gold before scanning electron microscopy (SEM), using a Jeol JFC-1200 fine coater (Jeol, Akishima, Japan). The tests were performed under high vacuum using an accelerating voltage of 5 kV. The SEM images of the fiber surfaces were recorded at 1000× and 2000× magnifications.

#### 2.3.4. Mechanical Properties of Fabrics Modified with Beeswax

Mechanical tests on fabric samples, before and after modification with beeswax, were carried out using a tensile testing machine, INSTRON Model 5944 (INSTRON, Norwood, MA, USA). The values of breaking force (F_max_) and elongation at break (ε_br_) were determined according to ISO13934-1 [28]. All fabric samples before testing were conditioned for at least 24 h at 20 °C and 65% relative humidity. Changes in the tensile strength of the textiles caused by the beeswax were evaluated by comparing the F_max_ values for the modified and unmodified fabric samples.

#### 2.3.5. Optical Properties of Fabrics Modified with Beeswax

Modification processes can change the optical characteristic of textiles and thus change their aesthetic features. The color changes of the beeswax-modified fabrics were determined according to EN ISO 105-J01 [29] using a double-beam spectrophotometer, V–670 UV–Vis–NIR (JASCO, Easton, MD, USA). The CIE Lab color system was used to calculate the total color changes of the fabric samples after the beeswax modification process. The following equation was used:

∆E = [(L − L_o_)^2^ + (a − a_o_)^2^ + (b − b_o_)^2^]^1/2,^ (1)

where L, a, b are the color coordinate values of the sample after modification, L_o_, a_o_, b_o_ are the color coordinate values of the sample before modification.

An increase in L means that the modified fabric sample is lighter than the control sample, while a decrease indicates that it is darker. An increase in the value of ‘a’ indicates that the fabric sample after modification is redder than the control, while a decrease means that the sample is greener. An increase in the value in ‘b’ indicates a greater degree of yellowness of the modified fabric sample, while a decrease in ‘b’ signifies an increase in blueness.

#### 2.3.6. Wettability and Hygroscopicity of Fabrics Modified with Beeswax

The wettability and capability of moisture sorption of fabrics are one of the most important properties for many applications. The high quality of clothing, bedding, towels is determined to a significant degree by the hygroscopicity of the textiles from which they are made.

Because of the hydrophobic properties of beeswax the fabrics finished with beeswax suspension can change their hydrophilic/hydrophobic characteristics. Changes in these characteristics caused by the beeswax were assessed by examining water contact angle and capability of moisture sorption for the fabric samples before and after modification.

The water contact angle values were measured by the sessile drop method [30]. Five measurements were made for each fabric sample using 1 μL distilled water drops.

The hygroscopicity of textiles were determined on the basis of equilibrium moisture content of fabric samples conditioned for at least 24 h at 20 °C and different relative air humidity (65% and 100%). The hygroscopicity (H) of sample was calculated by using the following formula:

H = [(W_m_ − W_0_)/W_0_] × 100%,

where W_m_—weight of the fabric sample conditioned at RH of 65% or 100% (in g), W_0_—weight of the fabric sample after drying to a constant mass (in g), In all the cases data from three repeated experiments were taken.

### 2.4. Estimation of the Antimicrobial Activity of Textiles Modified with Beeswax

#### 2.4.1. Microorganisms

To assess the antimicrobial activity of the textiles containing beeswax, we used five strains from pure culture collections: *Escherichia coli* 10,536, *Staphylococcus aureus* 6538, *Candida albicans* 10,231 and *Aspergillus niger* 16,404 from the American Type Culture Collection (ATCC); and *Bacillus subtilis* 01,644 from the National Collection of Agricultural and Industrial Microorganisms (NCAIM). The strains were selected based on taxonomic variety (gram-positive cocci, gram-negative rods, gram-positive bacilli, yeast and mold). The selected strains were also characterized by their varying ability to survive in the environment based on the production of either endospores (*B. subtilis*), spores (*A. niger*) or just vegetative cells (*E. coli, S. aureus* and *C. albicans*). A microorganism inoculum was prepared: bacteria and yeast colonies were transferred into 10 mL of TSB (Tryptic Soy Broth, Merck, Germany) and MEB medium (Malt Extract Broth, Merck, Germany), respectively and incubated at a temperature of 30 ± 2 °C for 24 h (bacteria), 27 ± 2 °C for 24 h (yeasts) and 72 h (molds). In the case of mold, colonies from MEA (Malt Extract Agar, Merck, Germany) slants were washed using 10 mL distilled water with 0.01% of Tween^®^80. The average density of the suspension ranged from 3.3 × 10^8^ to 9.5 × 10^9^ CFU/mL for bacteria; and from 1.10 × 10^7^ to 6.10 × 10^7^ CFU/mL for fungi.

#### 2.4.2. Assessment of Antimicrobial Activity

The antimicrobial activity of textiles with beeswax was measured using the AATCC 100-2004 modified quantity method [31].

The tests were conducted using samples of textiles (modified with beeswax fabrics 1 and 2; and without beeswax-control) with a surface area of 4 cm^2^ each. Next, 100 μL of inoculum of microorganisms was applied and the fabrics were placed in a climatic chamber (Binder, Germany) at 28 ± 2 ˚C with a relative humidity of 80%. The samples were collected immediately after applying the inoculum (t = 0 h) and after 24 h. The samples were then placed in 50 mL of sterile saline (0.85% NaCl) and shaken for 10 min (150 rev/min, shaker Elpin+, Lubawa, Poland) to wash out the microorganisms from the tested materials. Bacteria were incubated at 30 ± 2 °C for 24–48 h on TSA medium and fungi on MEA medium at 27 ± 2 °C for 3–5 days. The number of microorganisms (CFU/sample) was determined using the plate count method (dilutions from 10^0^ to 10^5^ in three repetitions). The tests were conducted in three independent repetitions for each type of fabric and time of incubation.

#### 2.4.3. Mathematical Calculations

We calculated the arithmetic means and standard deviations for the number of microorganisms on the surfaces of the materials tested. The antimicrobial effects of the fabrics were described using three parameters: (1) biocidal activity, (2) biostatic activity and (3) survival rate of the microorganisms on the tested fabrics and were calculated on the basis of literature [32,33]. The criteria for antimicrobial activity of the textiles containing beeswax were established using normative regulation for determining the biostatic and biocidal effects of disinfectants against bacteria and fungi according to EN 1276:2009 and EN 1650:2008 [34,35]. A value below 0.5 was accepted as low and meant a minimum threefold increase in the number of microorganisms. A value between 0.5 and 3 was classified as medium activity. A value of 3 or more was regarded as high and meant a thousand-fold or higher, increase in the number of microorganisms.

### 2.5. Statistical Analysis

Differences between the mechanical properties (tensile strength and elongation at break) of the beeswax containing fabrics and controls were analyzed using one-way Analysis of Variance (ANOVA) at a significance level *p* < 0.05. This method was also used to analyze differences between the number of microorganisms in the textiles with beeswax and control samples. All data were analyzed using the computer program Origin 6.1 (OriginLab, Northampton, MA, USA).

## 3. Results and Discussion

### 3.1. Analysis of the Chemical Composition of Beeswax

In order to characterize the lipid constituents of the beeswax, the ^109^AgNPET LDI MS, a soft ionization, derivatization-less qualitative method was used. The putative components of beeswax identified, based on the LipidMaps database, is presented in Table 2. Our analysis identified fatty acids, monoacyl esters, glyceride esters and more complex lipids such as sphingolipids, glycerophosphocholines, sterol lipids, glycerophosphoethanolamines, glycerophosphates, glycerophosphoglycerols and glycerophosphoserines.

The identified compounds have previously been described as substances in beeswax obtained from various countries [15,16,17].

### 3.2. Properties of the Modified Textiles

#### 3.2.1. SEM Analysis of the Distribution of Beeswax in the Fabric Structure

**Figure 1:**
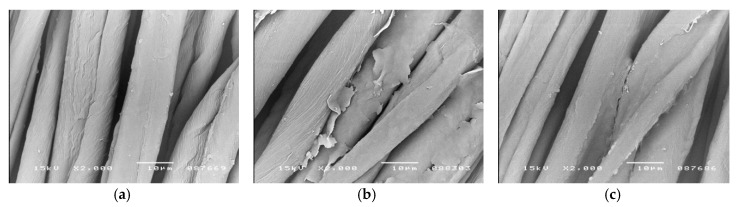
Scanning electron microscope (SEM) images of cotton fibers surface before and after fabric modification (magnification 2000×); (**a**)—Control sample (before modification); (**b**)—After impregnation with beeswax suspension and drying at room temperature; (**c**)—Sample b after heat treatment at 120 °C for 1 min.

**Figure 2:**
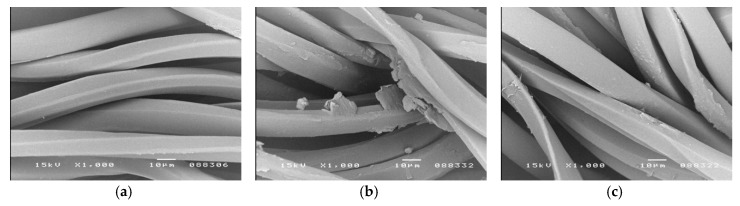
SEM images of polyester fibers surface before and after fabric modification (magnification 1000×); (**a**)—Control sample (before modification); (**b**)—After impregnation with beeswax suspension and drying at room temperature; (**c**)—Sample b after heat treatment at 120 °C for 1 min.

**Figure 3:**
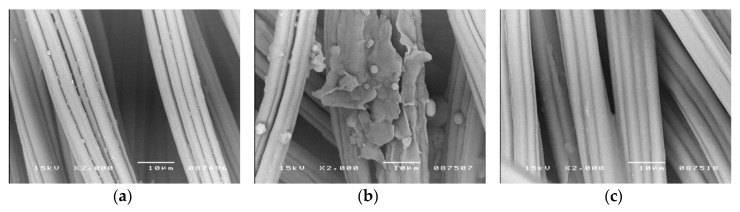
SEM images of viscose fibers surface before and after fabric modification (magnification 2000×); (**a**)—Control sample (before modification); (**b**)—After impregnation with beeswax suspension and drying at room temperature; (**c**)—sample B after heat treatment at 120 °C for 1 min.

Microscopic images (SEM) in Figure 1, Figure 2 and Figure 3 show the distribution of beeswax in the internal structure of fabrics at various stages of the modification process.

In fabric samples impregnated with beeswax and dried at room temperature, some fine beeswax particles of a few micrometers, in the form of thin flakes of irregular shapes and sizes, are visible on the surface of the fibers (Figure 1b, Figure 2b and Figure 3b).

In heat-treated fabric samples (at 120 °C for 1 min), these beeswax particles are no longer observed on the surface of the fibers. As a result of heating the fabric, when its temperature exceeds about 60 °C, the beeswax particles begins to melt and then the fabric temperature continues to rise up to around 120 °C and the beeswax particles “spilled” over the surface of the fibers. It should be assumed that in the case of cellulosic fibers (cotton and viscose), which are characterized by a porous structure, beeswax also penetrated the pores. In contrast, the compact, non-porous structure of polyester fibers would not allow the penetration of the beeswax into the interior of the fibers. In Figure 2c, a relatively even distribution of beeswax on the surface of the polyester fibers can be observed.

All SEM images show the characteristic morphological structure of the fiber surface, typical for cotton, polyester and viscose. The microscopic images of the surface of cotton, polyester and viscose fibers, after modification with beeswax (Figure 1c, Figure 2c and Figure 3c), show no visible differences in comparison with the unmodified fibers (Figure 1a, Figure 2a and Figure 3a). This indicates that the fabric modification process carried out with the beeswax suspension did not cause any changes to the morphological structure of the fibers.

#### 3.2.2. Tensile Strength of Fabrics Modified with Beeswax

The results in Table 3 indicate that both fabrics modified with beeswax have a very small reduction in breaking force (F_max_) compared to unmodified textiles. After the finishing process, the tensile strength of the polyester/cotton/viscose blended fabric (Fabric 1) decreased by less than 4%, while that of the polyester sample (Fabric 2) decreased by only about 2%. However, statistically significant differences were noted between both modified and control fabrics (*p* > 0.05).

All fabrics tested showed a trend towards increased elongation of the fibers due to the modification process (Table 3). It should be noted that the characteristic sign of fiber degradation (as well as of other polymeric materials), when they are subjected to destructive factors such as heat, certain biological factors, UV or gamma radiation, is a reduction in the elongation at break during tensile tests [36,37,38,39]. Thus, the values of the ε_br_ parameter given in Table 3 may indicate that the beeswax suspension used in the fabric modification process did not degrade the fibers.

The slight changes in the mechanical properties of the fabrics after the modification process were caused by the presence of beeswax in the internal structure of the fabrics (as well as on the surface of individual fibers). This may cause a decrease in the frictional forces between these fibers, which in turn may lead to some weakening of the tensile strength of the fabric and to an increase in elongation at break.

In general, the results obtained indicate that cellulose (the main component of cotton and viscose fibers) and polyester show similar high resistance to the fabric modification process. It is worth mentioning that the tensile strength of fabrics during some industrial processes (e.g., crease resistant and flame-retardant finishing of cotton fabrics) is reduced by up to 15–20% [37,38,39].

#### 3.2.3. Color Changes of Fabrics Caused by Beeswax Modification

The results in Table 4 show that for both the polyester and blended fabrics there were no significant changes in the color parameters between the samples before and after the beeswax modification process.

These are expected results, because the beeswax suspension applied to the fabrics has a slightly golden-yellow color. Application of this suspension on a cream colored textile product (Fabric 1) caused only a slight increase in its lightness (ΔL = 1.12), while the samples of dark brown polyester fabric (Fabric 2) became slightly darker (ΔL = −0.42).

The small values of Δa and Δb, from our colorimetric measurements, showed that for both tested fabrics there were no significant differences between the shade of color before and after the modification process. Based on these results, we conclude that applying beeswax to the fabrics caused only slight changes in their color, which are completely invisible to an inexperienced observer. This is confirmed by the values of total color change (ΔE) between the modified and control fabric samples, which are equal to 1.12 and 0.45 (Table 4), respectively.

#### 3.2.4. Hygroscopic Properties Changes of Fabrics Caused by Beeswax Modification

The results of the wettability and capability of moisture sorption of fabrics modified with beeswax are given in Table 5. The results of the contact angle test indicate that both fabric before modification showed excellent wettability. The water droplet deposited on the fabrics surface due to the high capillary action of fibers was immediately absorbed. The finishing process caused a very significant hydrophobization of the fabric surface. It has to be noticed that the difference between Fabric 1 and Fabric 2 is not significant, which is due to a similar amount of beeswax on the surface of both fabrics.

As anticipated, the polyester/cotton/viscose blended fabric (Fabric 1) showed a trend towards decreased hygroscopicity of the fibers due to the modification process. However, it should be emphasized that the deterioration of sorption capacity of this fabric is not drastic.

The amount of beeswax deposited on the fabric surface is greater than in the inner layers of the textile product. Thanks to this, the Fabric 1 has a hydrophobic surface and its inner layers (containing, among others cotton and viscose fibers) exhibit relatively high hygroscopicity. The results in Table 5 show that in the case of Fabric 2 (polyester) there were no significant changes in the wetting and sorption properties between the samples before and after the beeswax modification process.

### 3.3. Assessment of Antimicrobial Activity

**Figure 4:**
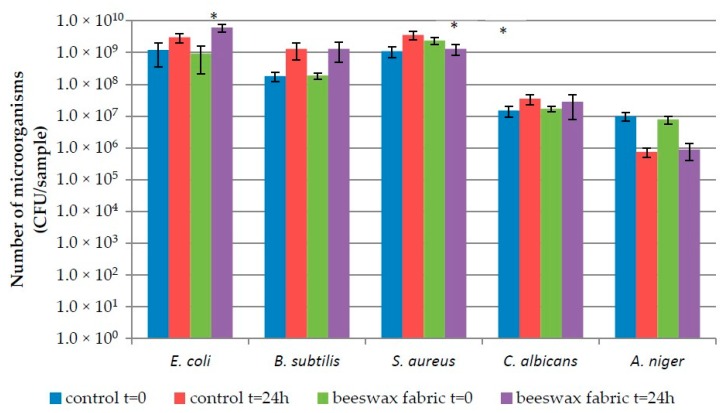
Number of microorganisms on Fabric 1; *—Statistically significant differences between number of microorganisms on control and beeswax-modified samples in the same time (one-way ANOVA, *p* < 0.05).

**Figure 5:**
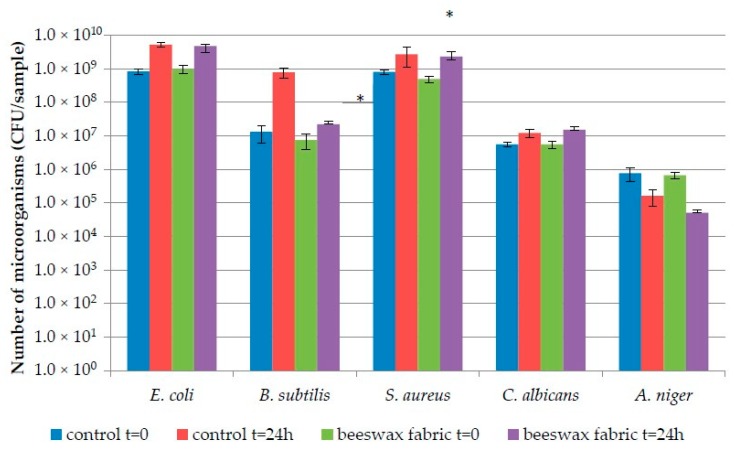
Number of microorganisms on Fabric 2. *—Statistically significant differences between number of microorganisms on control and beeswax-modified samples in the same time (one-way ANOVA, *p* < 0.05).

Microorganism numbers on control and beeswax containing fabrics, immediately after applying the inoculum of a given strain and following a 24 h incubation period, are shown in Figure 4 and Figure 5.

The number of bacteria on Fabric 1 at t = 0 ranged from 1.8 × 10^8^ CFU/sample (*B. subtilis*) to 1.2 × 10^9^ CFU/sample (*E. coli*) for the control fabric and from 1.9 × 10^8^ CFU/sample (*B. subtilis*) to 2.4 × 10^9^ CFU/sample (*S. aureus*) on the one modified with beeswax.

The number of fungi ranged from 9.9 × 10^6^ CFU/sample to 1.5 × 10^7^ CFU/sample on control fabric 1, while on the modified fabric, from 7.9 × 10^6^ CFU/sample to 1.7 × 10^7,^ CFU/sample (Figure 4). Statistically significant differences in microorganism numbers at t = 0 (*p* < 0.05) for the two types of fabrics (Fabrics 1 and 2) were recorded for *S. aureus* only.

Following incubation, microorganism numbers were in the range of 1.3 × 10^9^ CFU/sample (*B. subtilis*) − 3.6 × 10^9^ CFU/sample (*S. aureus*) for the control Fabric 1 and 1.3 × 10^9^ CFU/sample (*B. subtilis* and *S. aureus*) to 6.1 × 10^9^ CFU/sample (*E. coli*) for the beeswax-modified variant. The number of fungi after incubation was 7.5 × 10^5^ CFU/sample − 3.4 × 10^7^, CFU/sample and 8.9 × 10^5^ CFU/sample − 2.8 × 10^7,^ CFU/sample for the control and modified fabrics, respectively (Figure 4).

After incubation, statistically significant (*p* < 0.05) differences, between the variants of Fabric 1, were found for *E. coli* and *S. aureus*.

On Fabric 2 at t = 0, bacterial numbers were in the range of 1.3 × 10^7^ CFU/sample (*B. subtilis*) − 8.2 × 10^8^ CFU/sample (*E. coli*) on the control fabric and 7.4 × 10^6^ CFU/sample (*B. subtilis*) − 9.7 × 10^8^ CFU/sample (*E. coli*) on the beeswax-modified one. The number of fungi ranged from 7.6 × 10^5^ CFU/sample to 5.5 × 10^6,^ CFU/sample (control fabric) and from 6.6 × 10^5^ CFU/sample to 5.5 × 10^6,^ CFU/sample (modified fabric) (Figure 2). Statistically significant differences (*p* < 0.05) in microorganism numbers were recorded for the two variants of Fabric 2 (control and modified), for *S. aureus* only.

Following incubation, microorganism numbers on Fabric 2 control was 7.8 × 10^8^ CFU/sample (*B. subtilis*) − 2.7 × 10^9^ CFU/sample (*S. aureus*) and 2.2 × 10^7^ CFU/sample (*B. subtilis*) − 4.7×10^9^ CFU/sample (*E. coli*) on the modified sample. The number of fungi equaled 1.6 × 10^5^ CFU/sample − 1.5 × 10^7,^ CFU/sample for the control and 5.0 × 10^4^ CFU/sample − 1.5 × 10^7^, CFU/sample for the modified fabric (Figure 5).

Following incubation, statistically significant differences (*p* < 0.05) between the variants of Fabric 2 were only found for *B. subtilis.*

Antimicrobial effects of the beeswax-modified fabrics are described in Table 6. Microorganism survival rate on Fabric 1 ranged from 11.3% (*A. niger*) to 677.4% (*B. subtilis*), while on Fabric 2 from 7.6% (*A. niger*) to 484.5% (*E. coli*). Survival rates indicate that all strains tested can survive on fabrics modified with beeswax. Bacteria (with the exception of *S. aureus* on Fabric 1) had a higher survival rate, while yeast and mold lower.

Biostatic activity was higher for Fabric 2 (from −0.09 to 1.55) than for Fabric 1 (from −0.3 to 0.44). Thus Fabric 1 has low activity against all microorganisms tested, while Fabric 2 has low activity against *E. coli*, *S. aureus* and *C. albicans* and medium activity against *A. niger* and *B. subtilis*. In turn, biocidal activity ranged from −0.28 to 1.05 and from −0.76 to 1.18 for Fabrics 1 and 2, respectively. The highest biocidal activity (>1) was obtained for both fabric variants against *A. niger,* which means that beeswax-modified fabrics have medium activity against mold.

We believe that this study is the first attempt at endowing fabrics with antimicrobial properties using beeswax.

There are some similarities with the studies of Pinto et al. (2017), who tested the efficiency of Abeego (a commercially available food wrap) against representatives of different groups of microorganisms. Abeego consists of a fabric coated with beeswax, with minor components such as tree resin and oils. The authors demonstrated the antibacterial activity of Abeego against *Salmonella enteridis* and *Staphylococcus aureus*, which they attributed to all its ingredients (wax, resin, oil and fabric). They found no antifungal activity against *Saccharomyces cerevisiae* yeast [40].

Zhang and Xiao (2013) grafted polymeric guanidines including polyhexamethylene guanidine hydrochloride (PHGH) on beeswax latex beads, with or without amphoteric surfactant [41]. Novel beeswax latexes were utilized as a dual-functional paper additive, which improved both water-vapor resistance and antimicrobial activities of the paper [41]. The growth inhibition values of beeswax-modified PHGH were higher than 92%. By contrast, the beeswax latex without grafting of PHGH had no obvious effect on antimicrobial activity [42].

Data on the antimicrobial efficiency of beeswax solution, expressed as the zone of microorganism growth inhibition on an agar plate, can be found in the literature. This study, conducted by Ghanem et al. (2011), showed a higher efficiency of ethanolic extracts of beeswax against *Candida albicans* yeast and *S. aureus*, *S. epidermis*, *B. subtilis* and *Streptomyces pyogenes*, bacteria [26]. Similar, to the current study, they recorded lower efficiency against *E. coli* and gram-negative bacteria (*Proteus mirabilis*, *Salmonella typhimurium* and *Pseudomonas aeruginosa*). The authors did not test beeswax activity against molds [26].

Likewise, Kacániová et al. (2012) showed that, among five strains of bacteria, three strains of fungi and seven of yeast, the ones most sensitive to methanolic and ethanolic extracts of beeswax were *E. coli*, *A. niger* and *C. tropicalis* [25]. In the current work, *A. niger* mold was the most sensitive to the modified fabrics; however, no effect was seen against *E. coli.*

Published data on the antimicrobial activity of beeswax are not consistent with each other. Our results are difficult to compare with published literature, especially since the tests were carried out using a quantitative method. Our method allows microorganism CFU determination before and after contact with the modified fabric and compares these values to microorganism numbers on the control fabrics. Directly testing modified fabrics rather than only testing beeswax extracts has greater practical significance as the former are closer to practical applications of such fabrics. Moreover, many studies of antimicrobial activity of bee products did not show the chemical composition of the raw material used. As this is not standardized, it can be different in each experiment.

Beeswax is a natural product and the antimicrobial activity of fabrics modified with it can differ depending on a number of parameters. These include, the geographical region of beeswax origin, climatic conditions during the year of collection, health of the bees and the method of obtaining wax from the beehive.

## 4. Conclusions

The process of modifying polyester and polyester/cotton/viscose fabrics with beeswax did not result in changes to the morphological structure of the fibers nor in any visible changes to their color. Our impregnation method resulted in the uniform coating of the cellulosic and polyester fibers in both fabric types, which was confirmed by SEM analysis. Small but statistically significant, changes in mechanical properties of the fabrics (a small percentage decrease in the tensile strength and an increase in the elongation at break) were recorded. However, such changes were smaller than those that occur during some industrial processes like crease resistant and flame-retardant finishing of cotton and polyester/cotton fabrics.

We found low biostatic activity during our tests of antimicrobial effectiveness of the fabrics modified with beeswax. An exception was polyester fabric, which was biostatically active against *A. niger* mold and *B. subtilis* bacteria. In addition, both fabrics demonstrated medium biocidal activity (>1) against *A. niger* mold.

Our results indicate that the process of finishing fabrics using beeswax may endow them with antimold activity that can find a practical application. These fabrics can be used to manufacture textile products, for example, for the prophylaxis of skin mycoses in health and social care institutions, as well as for people engaged in a sport activity or with recurrent skin infections.

However, during the manufacture of such products, we recommend that each batch of beeswax undergoes tests to verify its antimicrobial activity, due to variabilities in the chemical composition of this material. Despite the beneficial properties of beeswax on human skin, described in the literature, it is important to verify whether fabrics modified with it also exhibit such positive effects.

## Figures and Tables

**Table 1:** Characteristics of tested fabrics.

Symbol	Raw Material	Mass Per Unit Area (g/m^2^)	Thickness, (mm)	Porosity (%)	Apparent Density (g/cm^3^)
Fabric 1	Polyester 40% Cotton 20%Viscose 40%	280	0.88	78	0.32
Fabric 2	Polyester 100%	164	0.36	67	0.45

Both the polyester and blended fabrics used in this study contained only dyes and no chemical finishing agents.

**Table 2:** Putative identification of beeswax components analyzed directly with ^109^AgNPET laser desorption/ionization mass spectrometry (LDI MS) based on LipidMaps database.

Compound Name ^1^	Formula	MW ^2^	Ion Type	Ion *m*/*z* ^3^	Δ (ppm)
Ethyl propionate	C_5_H_10_O_2_	102.0681	M+H	103.0754	6.4
FA(5:1)	C_5_H_8_O_2_	100.0524	M+Na	123.0416	2.0
Dimethylcyclohexane	C_8_H_16_	112.1252	M+Na	135.1144	8.3
Octadienal	C_8_H_12_O	124.0888	M+Na	147.0780	1.8
FA(16:1)	C_16_H_30_O_2_	254.2246	M+K	293.1877	8.7
Eicosatetraenoyl amine	C_20_H_33_NO	303.2562	M+H	304.2635	2.0
FA oxo(5:1/5:0/6:0)	C_16_H_26_O_3_	266.1882	M+K	305.1514	1.4
4′-Hydroxy-5,7,3′-trimethoxyflavan	C_18_H_20_O_5_	316.1311	M+H	317.1384	2.6
SP (3:0) sphingatrienine	C_18_H_33_NO_2_	295.2511	M+K	334.2140	3.9
FA hydroxy(18:1) 9-hydroxyoctadecenoic acid	C_18_H_34_O_3_	298.2508	M+K	337.2140	4.0
FA(16:1)	C_16_H_30_O_2_	254.2246	M+^109^Ag	363.1288	6.8
Ethyl tetradecanoate	C_16_H_32_O_2_	256.2402	M+^109^Ag	365.1444	0.2
PC(7:0) 1-heptanoyl-sn-glycero-3-phosphocholine	C_15_H_32_NO_7_P	369.1916	M+Na	392.1809	4.2
ST(5:0/2:0) 9,10-seco-5,7,10(19),16,23-cholestapentaene-3,25-diol	C_24_H_44_O_3_	380.329	M+K	419.2922	2.8
octacosaoctaenoic acid	C_28_H_40_O_2_	408.3028	M+K	447.2660	2.0
FA hydroxy,oxo(2:0) 9S,15S-dihydroxy-11-oxo-5Z,13E-prostadienoic acid 2-glyceryl ester	C_27_H_38_O_3_	410.2821	M+K	449.2453	5.4
Fv hydroxy,dimethoxy(9:1) 7,4′-Dihydroxy-8-lavandulyl-5,2′-dimethoxyflavanone	C_27_H_32_O_6_	452.2199	M+H	453.2272	6.0
PE (16:2) 1-hexadecyl-sn-glycero-3-phosphoethanolamine	C_21_H_46_NO_6_P	439.3063	M+K	478.2694	4.1
FA (26:0) hexacosenoic acid	C_26_H_50_O_2_	394.3811	M+^109^Ag	503.2853	6.5
PA(12:0/12:0)	C_27_H_53_O_8_P	536.3478	M+Na	559.3370	7.8
3-Hexaprenyl-4-hydroxybenzoic acid	C_37_H_54_O_3_	546.4073	M+Na	569.3965	8.8
PC (24:0) 1-tetracosanoyl-sn-glycero-3-phosphocholine	C_32_H_66_NO_7_P	607.4577	M+H	608.4650	9.3
FA (26:0/2:0) 1-(*O*-alpha-d-glucopyranosyl)-hexacosanediol	C_32_H_64_O_8_	576.4601	M+K	615.4233	1.3
PA(16:0/14:0)	C_33_H_65_O_8_P	620.4417	M+Na	643.4309	6.0
Cholest-5-en-3β-yl (7Z-hexadecenoate)	C_43_H_74_O_2_	622.5689	M+Na	645.5581	5.7
GL(8:0/8:0) 1-(8-(3)-ladderane-octanyl)-2-(8-(3)-ladderane-octanyl)-sn-glycerol	C_43_H_72_O_3_	636.5482	M+K	675.5113	8.3
PC(16:0/9:0(CHO))	C_33_H_64_NO_9_P	649.4319	M+K	688.3950	6.9
Tetracosanyl palmitoleate	C_40_H_78_O_2_	590.6002	M+^109^Ag	699.5044	6.5
PA(16:0/20:2)	C_39_H_73_O_8_P	700.5043	M+H	701.5116	1.3
PA(20:0/16:0)	C_39_H_77_O_8_P	704.5356	M+Na	727.5248	0.8
PG(15:0/18:3)	C_39_H_71_O_10_P	730.4785	M+H	731.4858	4.4
PG(15:1/18:1)	C_39_H_73_O_10_P	732.4941	M+H	733.5014	0.8
PA(18:3/22:1)	C_43_H_77_O_8_P	752.5356	M+H	753.5429	1.3
TG(12:0/12:0/18:3)(iso3)	C_45_H_80_O_6_	716.5955	M+K	755.5587	1.0
SM(d18:0/17:0)	C_40_H_83_N_2_O_6_P	718.5989	M+K	757.5620	1.5
PG(20:2/15:1)	C_41_H_75_O_10_P	758.5098	M+H	759.5171	6.5
MGDG(18:0/18:2) di-(octadecatrienoyl)-3- *O*-β-d-galactosyl-sn-glycerol	C_45_H_82_O_10_	782.5908	M+H	783.5981	0.1
SM(d16:1/24:0)	C_45_H_91_N_2_O_6_P	786.6615	M+H	787.6688	3.6
PS(P-16:0/14:1)	C_36_H_68_NO_9_P	689.4632	M+^109^Ag	798.3674	0.1
PG(*O*-16:0/16:0)	C_38_H_77_O_9_P	708.5305	M+^109^Ag	817.4347	5.8
3-*O*-(6′-*O*-hexadecanoyl-beta-d-glucopyranosyl)-stigmast-5-en-3beta-ol	C_51_H_90_O_7_	814.6687	M+Na	837.6579	5.9
3-*O*-(6′-*O*-(9Z,12Z-octadecadienoyl)-beta-d-glucopyranosyl)-stigmast-5-en-3beta-ol	C_53_H_90_O_7_	838.6687	M+H	839.6759	3.1
CoA(22:1)	C_43_H_76_N_7_O_17_P_3_S	1087.4231	M+H	1088.4304	9.0
Galbeta1-3GalNAcbeta1-4(KDNalpha2-3)Galbeta1-4Glcbeta-Cer(d18:1/20:0)	C_73_H_132_N_2_O_31_	1532.8814	M+K	1571.8446	0.9
Galalpha1-3(Fucalpha1-2)Galbeta1-4GlcNAcbeta1-3Galbeta1-4GlcNAcbeta1-3Galbeta1-4Glcbeta-Cer(d18:1/18:0)	C_88_H_157_N_3_O_42_	1928.0242	M+K	1966.9873	4.2

^1^ Other matching isomeric compounds are shown in Supplementary Materials (Table S1); ^2^ ion monoisotopic mass; ^3^ calculated monoisotopic mass.

**Table 3:** Tensile strength (F_max_) and elongation at break (ε_br_) of fabrics after modification with beeswax.

Symbol	Raw Material (%)	Beeswax Content in Fabric (%)	F_max_ (N)	ε_br_ (%)	Relative Decrease in F_max_ Values Caused by Fabric Modification (%)
Fabric 1	Polyester 40 Cotton 20 Viscose 40	0 (control)	360.9 ± 3.8	14.8 ± 0.4	3.7
		3.00	347.4 ± 3.5 *	16.4 ± 0.6 *	
Fabric 2	Polyester 100%	0 (control)	484.4 ± 5.8	54.8 ± 1.3	2.4
		2.25	472.7 ± 4.4 *	57.3 ± 0.8 *	

* statistically significant differences between control and beeswax-modified samples (one-way ANOVA, *p* < 0.05).

**Table 4:** Optical parameters of fabrics after modification with beeswax.

Symbol	Beeswax Content (%)	L	a	b	ΔL	Δa	Δb	ΔE
Fabric 1	0 (control)	84.73	1.99	12.91	1.12	0.01	0.05	1.12
	3.00	85.85	2.00	12.96				
Fabric 2	0 (control)	18.67	4.37	1.09	−0.42	0.15	0.06	0.45
	2.25	18.25	4.52	1.16				

**Table 5:** Water contact angle (WCA) and hygroscopicity (H) (at different relative air humidity) of fabrics after modification with beeswax.

Symbol	Beeswax Content (%)	WCA (°)	H (%) at RH 65%	H (%) at RH 100%
Fabric 1	0 (control)	0 *	6.8 ± 0.2	16.5 ± 0.5
	3.00	129.4 ± 3.3	5.0 ± 0.3	13.4 ± 0.7
Fabric 2	0 (control)	0 *	0.5 ± 0.1	2.1 ± 0.2
	2.25	112.2 ± 4.1	0.6 ± 0.1	2.0 ± 0.2

*—Measurements were not possible due to the immediate complete absorption of the water drop.

**Table 6:** Antimicrobial activity of beeswax-modified fabrics.

Microorganism		Survival Rate (%)		Biostatic Activity		Biocidal Activity	
		Fabric 1	Fabric 2	Fabric 1	Fabric 2	Fabric 1	Fabric 2
Bacteria	*Escherichia coli*	653.3	484.5	−0.3	0.04	−0.7	−0.76
	*Bacillus subtilis*	677.4	297.3	−0.02	1.55	−0.9	−0.23
	*Staphylococcus aureus*	56.2	469.4	0.44	0.07	−0.7	−0.46
Fungi	*Candida albicans*	166.2	272.7	0.10	−0.09	−0.28	−0.44
	*Aspergillus niger*	11.3	7.6	0.07	0.51	1.05	1.18

## Supplementary Materials

The following are available online at https://www.mdpi.com/2073-4360/12/2/344/s1, Table S1: Putative identification of metabolites in beeswax based on Lipid Maps database.
