# Supplementary material for: Beeswax-Modified Textiles: Method of Preparation and Assessment of Antimicrobial Properties
Source: Polymers (Basel). 2020 Feb 5;12(2):344. doi: 10.3390/polym12020344 (PMC7077388; doi:10.3390/polym12020344)
Supplement: Supplementary file 1 [file polymers-12-00344-s001.pdf]

Table S1. Putative identification of metabolites in beeswax based on Lipidimaps database

MS search for 103.0747 *m/z*

Compound	Formula	Compound MW	Adduct type	Adduct *m/z* calc	Delta [ppm]	Database
Ethyl propionate	$C_5H_{10}O_2$	102.0681	M+H	103.0754	-6.4	Lipidmaps
Methyl butyrate	$C_5H_{10}O_2$	102.0681	M+H	103.0754	-6.4	Lipidmaps
2-hydroxy-pentan-3-one	$C_5H_{10}O_2$	102.0681	M+H	103.0754	-6.4	Lipidmaps
3-Hydroxypentan-2-one	$C_5H_{10}O_2$	102.0681	M+H	103.0754	-6.4	Lipidmaps

MS search for 123.0419

[FA methyl(4:1)] 2-methyl-2E-butenoic acid	$C_5H_8O_2$	100.0524	M+Na	123.0416	2	Lipidmaps
[FA methyl(4:1)] 3-methyl-3-butenoic acid	$C_5H_8O_2$	100.0524	M+Na	123.0416	2	Lipidmaps
[FA (5:1)] 2-pentenoic acid	$C_5H_8O_2$	100.0524	M+Na	123.0416	2	Lipidmaps
[FA (5:1)] 3-pentenoic acid	$C_5H_8O_2$	100.0524	M+Na	123.0416	2	Lipidmaps
[FA (5:1)] 4-pentenoic acid	$C_5H_8O_2$	100.0524	M+Na	123.0416	2	Lipidmaps
Ethyl 2E-propenoate	$C_5H_8O_2$	100.0524	M+Na	123.0416	2	Lipidmaps
Gamma-valerolactone	$C_5H_8O_2$	100.0524	M+Na	123.0416	2	Lipidmaps

MS search for 123.0419 *m/z*

Compound	Formula	Compound MW	Adduct type	Adduct *m/z* calc	Delta [ppm]	Database
[FA methyl(4:1)] 2-methyl-2E-butenoic acid	$C_5H_8O_2$	100.0524	M+Na	123.0416	2	Lipidmaps
[FA methyl(4:1)] 3-methyl-3-butenoic acid	$C_5H_8O_2$	100.0524	M+Na	123.0416	2	Lipidmaps
[FA (5:1)] 2-pentenoic acid	$C_5H_8O_2$	100.0524	M+Na	123.0416	2	Lipidmaps
[FA (5:1)] 3-pentenoic acid	$C_5H_8O_2$	100.0524	M+Na	123.0416	2	Lipidmaps
[FA (5:1)] 4-pentenoic acid	$C_5H_8O_2$	100.0524	M+Na	123.0416	2	Lipidmaps
Ethyl 2E-propenoate	$C_5H_8O_2$	100.0524	M+Na	123.0416	2	Lipidmaps
Gamma-valerolactone	$C_5H_8O_2$	100.0524	M+Na	123.0416	2	Lipidmaps

MS search for 135.1133 *m/z*

Compound	Formula	Compound MW	Adduct type	Adduct *m/z* calc	Delta [ppm]	Database
3-methyl-1-heptene	C_8H_{16}	112.1252	M+Na	135.1144	-8.3	Lipidmaps
Cis-1,2-dimethylcyclohexane	C_8H_{16}	112.1252	M+Na	135.1144	-8.3	Lipidmaps

Trans-1,2-dimethylcyclohexane	C_8H_{16}	112.1252	M+Na	135.1144	-8.3	Lipidmaps
1,4-dimethylcyclohexane	C_8H_{16}	112.1252	M+Na	135.1144	-8.3	Lipidmaps
Ethylcyclohexane	C_8H_{16}	112.1252	M+Na	135.1144	-8.3	Lipidmaps

MS search for 147.0783 *m/z*

**Compound**	**Formula**	**Compound MW**	**Adduct type**	**Adduct *m/z* calc**	**Delta [ppm]**	**Database**
[FA (8:2)] 2,4-octadienal	$C_8H_{12}O$	124.0888	M+Na	147.078	1.8	Lipidmaps
[FA (8:2)] 5,7-octadienal	$C_8H_{12}O$	124.0888	M+Na	147.078	1.8	Lipidmaps
2,4-Dimethyl-2E,4E-hexadienal	$C_8H_{12}O$	124.0888	M+Na	147.078	1.8	Lipidmaps
2E,7-Octadienal	$C_8H_{12}O$	124.0888	M+Na	147.078	1.8	Lipidmaps
2E,6E-Octadienal	$C_8H_{12}O$	124.0888	M+Na	147.078	1.8	Lipidmaps
2E,4Z-Octadienal	$C_8H_{12}O$	124.0888	M+Na	147.078	1.8	Lipidmaps
2E,6Z-Octadienal	$C_8H_{12}O$	124.0888	M+Na	147.078	1.8	Lipidmaps
3E,5E-Octadien-2-one	$C_8H_{12}O$	124.0888	M+Na	147.078	1.8	Lipidmaps
6-Methyl-3E,5-heptadien-2-one	$C_8H_{12}O$	124.0888	M+Na	147.078	1.8	Lipidmaps

MS search for 293.1903 *m/z*

**Compound**	**Formula**	**Compound MW**	**Adduct type**	**Adduct *m/z* calc**	**Delta [ppm]**	**Database**
[FA dimethyl(14:0)] 2,4-dimethyl-2E-tetradecenoic acid	$C_{16}H_{30}O_2$	254.2246	M+K	293.1877	8.7	Lipidmaps
[FA methyl(15:0)] 14-methyl-4-pentadecenoic acid	$C_{16}H_{30}O_2$	254.2246	M+K	293.1877	8.7	Lipidmaps
[FA (6:2/10:0)] 2-hexyl-2-decenoic acid	$C_{16}H_{30}O_2$	254.2246	M+K	293.1877	8.7	Lipidmaps
[FA methyl(5:2/10:0)] 6-isopentyl-9-methyl-5-decenoic acid	$C_{16}H_{30}O_2$	254.2246	M+K	293.1877	8.7	Lipidmaps
[FA (16:1)] 7-hexadecenoic acid	$C_{16}H_{30}O_2$	254.2246	M+K	293.1877	8.7	Lipidmaps
[FA (16:1)] 10Z-hexadecenoic acid	$C_{16}H_{30}O_2$	254.2246	M+K	293.1877	8.7	Lipidmaps
[FA (16:1)] 10-hexadecenoic acid	$C_{16}H_{30}O_2$	254.2246	M+K	293.1877	8.7	Lipidmaps
[FA (16:1)] 11-hexadecenoic acid	$C_{16}H_{30}O_2$	254.2246	M+K	293.1877	8.7	Lipidmaps
[FA (16:1)] 11Z-hexadecenoic acid	$C_{16}H_{30}O_2$	254.2246	M+K	293.1877	8.7	Lipidmaps
[FA (16:1)] 13-hexadecenoic acid	$C_{16}H_{30}O_2$	254.2246	M+K	293.1877	8.7	Lipidmaps
[FA (16:1)] 13Z-hexadecenoic acid	$C_{16}H_{30}O_2$	254.2246	M+K	293.1877	8.7	Lipidmaps
[FA (16:1)] 2Z-hexadecenoic acid	$C_{16}H_{30}O_2$	254.2246	M+K	293.1877	8.7	Lipidmaps
[FA (16:1)] 3E-hexadecenoic acid	$C_{16}H_{30}O_2$	254.2246	M+K	293.1877	8.7	Lipidmaps
[FA (16:1)] 6Z-hexadecenoic acid	$C_{16}H_{30}O_2$	254.2246	M+K	293.1877	8.7	Lipidmaps
Hexadec-7Z-enoic acid	$C_{16}H_{30}O_2$	254.2246	M+K	293.1877	8.7	Lipidmaps
16:1(5Z)	$C_{16}H_{30}O_2$	254.2246	M+K	293.1877	8.7	Lipidmaps
15:1(4)(13Me)	$C_{16}H_{30}O_2$	254.2246	M+K	293.1877	8.7	Lipidmaps
16:1(4)	$C_{16}H_{30}O_2$	254.2246	M+K	293.1877	8.7	Lipidmaps

10E-Tetradecenyl acetate	$C_{16}H_{30}O_2$	254.2246	M+K	293.1877	8.7	Lipidmaps
11E-Tetradecenyl acetate	$C_{16}H_{30}O_2$	254.2246	M+K	293.1877	8.7	Lipidmaps
12E-Tetradecenyl acetate	$C_{16}H_{30}O_2$	254.2246	M+K	293.1877	8.7	Lipidmaps
3E-Tetradecenyl acetate	$C_{16}H_{30}O_2$	254.2246	M+K	293.1877	8.7	Lipidmaps
5E-Tetradecenyl acetate	$C_{16}H_{30}O_2$	254.2246	M+K	293.1877	8.7	Lipidmaps
6E-Tetradecenyl acetate	$C_{16}H_{30}O_2$	254.2246	M+K	293.1877	8.7	Lipidmaps
7E-Tetradecenyl acetate	$C_{16}H_{30}O_2$	254.2246	M+K	293.1877	8.7	Lipidmaps
8E-Tetradecenyl acetate	$C_{16}H_{30}O_2$	254.2246	M+K	293.1877	8.7	Lipidmaps
9E-Tetradecenyl acetate	$C_{16}H_{30}O_2$	254.2246	M+K	293.1877	8.7	Lipidmaps
10Z-Tetradecenyl acetate	$C_{16}H_{30}O_2$	254.2246	M+K	293.1877	8.7	Lipidmaps
11Z-Tetradecenyl acetate	$C_{16}H_{30}O_2$	254.2246	M+K	293.1877	8.7	Lipidmaps
12Z-Tetradecenyl acetate	$C_{16}H_{30}O_2$	254.2246	M+K	293.1877	8.7	Lipidmaps
3Z-Tetradecenyl acetate	$C_{16}H_{30}O_2$	254.2246	M+K	293.1877	8.7	Lipidmaps
5Z-Tetradecenyl acetate	$C_{16}H_{30}O_2$	254.2246	M+K	293.1877	8.7	Lipidmaps
6Z-Tetradecenyl acetate	$C_{16}H_{30}O_2$	254.2246	M+K	293.1877	8.7	Lipidmaps
7Z-Tetradecenyl acetate	$C_{16}H_{30}O_2$	254.2246	M+K	293.1877	8.7	Lipidmaps
8Z-Tetradecenyl acetate	$C_{16}H_{30}O_2$	254.2246	M+K	293.1877	8.7	Lipidmaps
9Z-Tetradecenyl acetate	$C_{16}H_{30}O_2$	254.2246	M+K	293.1877	8.7	Lipidmaps
Ethyl 7E-tetradecenoate	$C_{16}H_{30}O_2$	254.2246	M+K	293.1877	8.7	Lipidmaps
Ethyl 9E-tetradecenoate	$C_{16}H_{30}O_2$	254.2246	M+K	293.1877	8.7	Lipidmaps
Ethyl 9Z-tetradecenoate	$C_{16}H_{30}O_2$	254.2246	M+K	293.1877	8.7	Lipidmaps
Dodecyl 2E-butenoate	$C_{16}H_{30}O_2$	254.2246	M+K	293.1877	8.7	Lipidmaps
(Z)-7-Dodecenyl butyrate	$C_{16}H_{30}O_2$	254.2246	M+K	293.1877	8.7	Lipidmaps
Vittatalactone	$C_{16}H_{30}O_2$	254.2246	M+K	293.1877	8.7	Lipidmaps
15R-Hexadecanolide	$C_{16}H_{30}O_2$	254.2246	M+K	293.1877	8.7	Lipidmaps
16-Hexadecanolide	$C_{16}H_{30}O_2$	254.2246	M+K	293.1877	8.7	Lipidmaps
Delta-hexadecalactone	$C_{16}H_{30}O_2$	254.2246	M+K	293.1877	8.7	Lipidmaps

MS search for 304.2641 *m/z*

**Compound**	**Formula**	**Compound MW**	**Adduct type**	**Adduct *m/z* calc**	**Delta [ppm]**	**Database**
[FA (20:4)] 5Z,8Z,11Z,14Z-eicosatetraenoyl amine	$C_{20}H_{33}NO$	303.2562	M+H	304.2635	2	Lipidmaps
5Z,8Z,11Z,14Z-eicosatetraenoyl amine	$C_{20}H_{33}NO$	303.2562	M+H	304.2635	2	Lipidmaps

MS search for 305.1518 *m/z*

**Compound**	**Formula**	**Compound MW**	**Adduct type**	**Adduct *m/z* calc**	**Delta [ppm]**	**Database**
[FA hydroxy(16:3)] 8R-hydroxy-4Z,6E,10Z-hexadecatrienoic acid	$C_{16}H_{26}O_3$	266.1882	M+K	305.1514	1.4	Lipidmaps
[FA oxo(5:1/5:0/6:0)] (1R,2R)-3-oxo-2-(2'Z-pentenyl)cyclopentanehexanoic acid	$C_{16}H_{26}O_3$	266.1882	M+K	305.1514	1.4	Lipidmaps

[FA oxo(5:1/5:0/6:0)] (1S,2S)-3-oxo-2-(2'Z-pentenyl)cyclopentanehexanoic acid	$C_{16}H_{26}O_3$	266.1882	M+K	305.1514	1.4	Lipidmaps

MS search for 317.1392 *m/z*

Compound	Formula	Compound MW	Adduct type	Adduct *m/z* calc	Delta [ppm]	Database
4-Hydroxy-5,7,4'-trimethoxyflavan	$C_{18}H_{20}O_5$	316.1311	M+H	317.1384	2.6	Lipidmaps
2-(3,5-Dimethoxyphenyl)-3,4-dihydro-7-methoxy-2H-1-benzopyran-5-ol	$C_{18}H_{20}O_5$	316.1311	M+H	317.1384	2.6	Lipidmaps
(2S)-4'-Hydroxy-5,7,3'-trimethoxyflavan	$C_{18}H_{20}O_5$	316.1311	M+H	317.1384	2.6	Lipidmaps
[Fv Trihydroxy,methoxy,dimethy] 4,2',6'-Trihydroxy-4'-methoxy-3',5'-dimethyldihydrochalcone	$C_{18}H_{20}O_5$	316.1311	M+H	317.1384	2.6	Lipidmaps
[Fv Hydroxy,trimethox] 2'-Hydroxy-4,4',6'-trimethoxydihydrochalcone	$C_{18}H_{20}O_5$	316.1311	M+H	317.1384	2.6	Lipidmaps
[Fv Hydroxy,trimethox] 2'-Hydroxy-3',4',6'-trimethoxydihydrochalcone	$C_{18}H_{20}O_5$	316.1311	M+H	317.1384	2.6	Lipidmaps
[Fv] Loureirin B	$C_{18}H_{20}O_5$	316.1311	M+H	317.1384	2.6	Lipidmaps
[Fv Hydroxy,trimethox] 2-Hydroxy-3,4,6-trimethoxydihydrochalcone	$C_{18}H_{20}O_5$	316.1311	M+H	317.1384	2.6	Lipidmaps
[Fv Hydroxy,trimethox] 2-Hydroxy-4,5,6-trimethoxydihydrochalcone	$C_{18}H_{20}O_5$	316.1311	M+H	317.1384	2.6	Lipidmaps

MS search for 334.2156 *m/z*

Compound	Formula	Compound MW	Adduct type	Adduct *m/z* calc	Delta [ppm]	Database
[SP (3:0)] sphinga-4E,8E,10E-trienine	$C_{18}H_{33}NO_2$	295.2511	M+K	334.2143	3.9	Lipidmaps

MS search for 337.2126 *m/z*

Compound	Formula	Compound MW	Adduct type	Adduct *m/z* calc	Delta [ppm]	Database
9-hydroxy-12Z-octadecenoic acid	$C_{18}H_{34}O_3$	298.2508	M+K	337.214	-4	Lipidmaps
12-hydroxy-9E-octadecenoic acid	$C_{18}H_{34}O_3$	298.2508	M+K	337.214	-4	Lipidmaps
12-hydroxy-10E-octadecenoic acid	$C_{18}H_{34}O_3$	298.2508	M+K	337.214	-4	Lipidmaps
9-hydroxy-10Z-octadecenoic acid	$C_{18}H_{34}O_3$	298.2508	M+K	337.214	-4	Lipidmaps
9-hydroxy-12-octadecenoic acid	$C_{18}H_{34}O_3$	298.2508	M+K	337.214	-4	Lipidmaps

17-hydroxy-9Z-octadecenoic acid	$C_{18}H_{34}O_3$	298.2508	M+K	337.214	-4	Lipidmaps
18-hydroxy-9Z-octadecenoic acid	$C_{18}H_{34}O_3$	298.2508	M+K	337.214	-4	Lipidmaps
12R-hydroxy-9Z-octadecenoic acid	$C_{18}H_{34}O_3$	298.2508	M+K	337.214	-4	Lipidmaps
5-hydroxy-2-octadecenoic acid	$C_{18}H_{34}O_3$	298.2508	M+K	337.214	-4	Lipidmaps
8-hydroxy-9-octadecenoic acid	$C_{18}H_{34}O_3$	298.2508	M+K	337.214	-4	Lipidmaps
8R-hydroxy-9Z-octadecenoic acid	$C_{18}H_{34}O_3$	298.2508	M+K	337.214	-4	Lipidmaps
9R-hydroxy-10E-octadecenoic acid	$C_{18}H_{34}O_3$	298.2508	M+K	337.214	-4	Lipidmaps
9-hydroxy-10E-octadecenoic acid	$C_{18}H_{34}O_3$	298.2508	M+K	337.214	-4	Lipidmaps
9R-hydroxy-12E-octadecenoic acid	$C_{18}H_{34}O_3$	298.2508	M+K	337.214	-4	Lipidmaps
9R-hydroxy-12Z-octadecenoic acid	$C_{18}H_{34}O_3$	298.2508	M+K	337.214	-4	Lipidmaps
10-hydroxy-8-octadecenoic acid	$C_{18}H_{34}O_3$	298.2508	M+K	337.214	-4	Lipidmaps
10R-hydroxy-8E-octadecenoic acid	$C_{18}H_{34}O_3$	298.2508	M+K	337.214	-4	Lipidmaps
11-hydroxy-9-octadecenoic acid	$C_{18}H_{34}O_3$	298.2508	M+K	337.214	-4	Lipidmaps
12R-hydroxy-9E-octadecenoic acid	$C_{18}H_{34}O_3$	298.2508	M+K	337.214	-4	Lipidmaps
12S-hydroxy-9E-octadecenoic acid	$C_{18}H_{34}O_3$	298.2508	M+K	337.214	-4	Lipidmaps
12S-hydroxy-9Z-octadecenoic acid	$C_{18}H_{34}O_3$	298.2508	M+K	337.214	-4	Lipidmaps
3-oxo-octadecanoic acid	$C_{18}H_{34}O_3$	298.2508	M+K	337.214	-4	Lipidmaps
4-oxo-octadecanoic acid	$C_{18}H_{34}O_3$	298.2508	M+K	337.214	-4	Lipidmaps
5-oxo-octadecanoic acid	$C_{18}H_{34}O_3$	298.2508	M+K	337.214	-4	Lipidmaps
6-oxo-octadecanoic acid	$C_{18}H_{34}O_3$	298.2508	M+K	337.214	-4	Lipidmaps
7-oxo-octadecanoic acid	$C_{18}H_{34}O_3$	298.2508	M+K	337.214	-4	Lipidmaps
9-oxo-octadecanoic acid	$C_{18}H_{34}O_3$	298.2508	M+K	337.214	-4	Lipidmaps
10-oxo-octadecanoic acid	$C_{18}H_{34}O_3$	298.2508	M+K	337.214	-4	Lipidmaps
16-methyl-10-oxo-heptadecanoic acid	$C_{18}H_{34}O_3$	298.2508	M+K	337.214	-4	Lipidmaps
2-methyl-4-oxo-heptadecanoic acid	$C_{18}H_{34}O_3$	298.2508	M+K	337.214	-4	Lipidmaps
11-oxo-octadecanoic acid	$C_{18}H_{34}O_3$	298.2508	M+K	337.214	-4	Lipidmaps
12-oxo-octadecanoic acid	$C_{18}H_{34}O_3$	298.2508	M+K	337.214	-4	Lipidmaps
13-oxo-octadecanoic acid	$C_{18}H_{34}O_3$	298.2508	M+K	337.214	-4	Lipidmaps
14-oxo-octadecanoic acid	$C_{18}H_{34}O_3$	298.2508	M+K	337.214	-4	Lipidmaps
15-oxo-octadecanoic acid	$C_{18}H_{34}O_3$	298.2508	M+K	337.214	-4	Lipidmaps
16-oxo-octadecanoic acid	$C_{18}H_{34}O_3$	298.2508	M+K	337.214	-4	Lipidmaps
17-oxo-octadecanoic acid	$C_{18}H_{34}O_3$	298.2508	M+K	337.214	-4	Lipidmaps
2-oxo-octadecanoic acid	$C_{18}H_{34}O_3$	298.2508	M+K	337.214	-4	Lipidmaps
8-oxo-octadecanoic acid	$C_{18}H_{34}O_3$	298.2508	M+K	337.214	-4	Lipidmaps
6R,7S-epoxy-octadecanoic acid	$C_{18}H_{34}O_3$	298.2508	M+K	337.214	-4	Lipidmaps

[FA hydroxy(18:1)] 9-hydroxy-12Z-octadecenoic acid	$C_{18}H_{34}O_3$	298.2508	M+K	337.214	-4	Lipidmaps
[FA hydroxy(18:1)] 12-hydroxy-9Z-octadecenoic acid	$C_{18}H_{34}O_3$	298.2508	M+K	337.214	-4	Lipidmaps
[FA hydroxy(18:1)] 12-hydroxy-9E-octadecenoic acid	$C_{18}H_{34}O_3$	298.2508	M+K	337.214	-4	Lipidmaps
[FA hydroxy(18:1)] 12-hydroxy-10E-octadecenoic acid	$C_{18}H_{34}O_3$	298.2508	M+K	337.214	-4	Lipidmaps
[FA hydroxy(18:1)] 9-hydroxy-10Z-octadecenoic acid	$C_{18}H_{34}O_3$	298.2508	M+K	337.214	-4	Lipidmaps
[FA hydroxy(18:1)] 9-hydroxy-12-octadecenoic acid	$C_{18}H_{34}O_3$	298.2508	M+K	337.214	-4	Lipidmaps
[FA hydroxy(18:1)] 17-hydroxy-9Z-octadecenoic acid	$C_{18}H_{34}O_3$	298.2508	M+K	337.214	-4	Lipidmaps
[FA hydroxy(18:1)] 18-hydroxy-9Z-octadecenoic acid	$C_{18}H_{34}O_3$	298.2508	M+K	337.214	-4	Lipidmaps
[FA hydroxy(18:1)] 5-hydroxy-2-octadecenoic acid	$C_{18}H_{34}O_3$	298.2508	M+K	337.214	-4	Lipidmaps
[FA hydroxy(18:1)] 8-hydroxy-9-octadecenoic acid	$C_{18}H_{34}O_3$	298.2508	M+K	337.214	-4	Lipidmaps
[FA hydroxy(18:1)] 8R-hydroxy-9Z-octadecenoic acid	$C_{18}H_{34}O_3$	298.2508	M+K	337.214	-4	Lipidmaps
[FA hydroxy(18:1)] 9R-hydroxy-10E-octadecenoic acid	$C_{18}H_{34}O_3$	298.2508	M+K	337.214	-4	Lipidmaps
[FA hydroxy(18:1)] 9-hydroxy-10E-octadecenoic acid	$C_{18}H_{34}O_3$	298.2508	M+K	337.214	-4	Lipidmaps
[FA hydroxy(18:1)] 9R-hydroxy-12E-octadecenoic acid	$C_{18}H_{34}O_3$	298.2508	M+K	337.214	-4	Lipidmaps
[FA hydroxy(18:1)] 9R-hydroxy-12Z-octadecenoic acid	$C_{18}H_{34}O_3$	298.2508	M+K	337.214	-4	Lipidmaps
[FA hydroxy(18:1)] 10-hydroxy-8-octadecenoic acid	$C_{18}H_{34}O_3$	298.2508	M+K	337.214	-4	Lipidmaps
[FA hydroxy(18:1)] 10R-hydroxy-8E-octadecenoic acid	$C_{18}H_{34}O_3$	298.2508	M+K	337.214	-4	Lipidmaps
[FA hydroxy(18:1)] 11-hydroxy-9-octadecenoic acid	$C_{18}H_{34}O_3$	298.2508	M+K	337.214	-4	Lipidmaps
[FA hydroxy(18:1)] 12R-hydroxy-9E-octadecenoic acid	$C_{18}H_{34}O_3$	298.2508	M+K	337.214	-4	Lipidmaps
[FA hydroxy(18:1)] 12S-hydroxy-9E-octadecenoic acid	$C_{18}H_{34}O_3$	298.2508	M+K	337.214	-4	Lipidmaps
[FA hydroxy(18:1)] 12S-hydroxy-9Z-octadecenoic acid	$C_{18}H_{34}O_3$	298.2508	M+K	337.214	-4	Lipidmaps
8Z-decen-4,6-diynoic acid	$C_{18}H_{34}O_3$	298.2508	M+K	337.214	-4	Lipidmaps
[FA oxo(18:0)] 4-oxo-octadecanoic acid	$C_{18}H_{34}O_3$	298.2508	M+K	337.214	-4	Lipidmaps
[FA oxo(18:0)] 5-oxo-octadecanoic acid	$C_{18}H_{34}O_3$	298.2508	M+K	337.214	-4	Lipidmaps
[FA oxo(18:0)] 6-oxo-octadecanoic acid	$C_{18}H_{34}O_3$	298.2508	M+K	337.214	-4	Lipidmaps
[FA oxo(18:0)] 7-oxo-octadecanoic acid	$C_{18}H_{34}O_3$	298.2508	M+K	337.214	-4	Lipidmaps
[FA oxo(18:0)] 9-oxo-octadecanoic acid	$C_{18}H_{34}O_3$	298.2508	M+K	337.214	-4	Lipidmaps

[FA oxo(18:0)] 10-oxo-octadecanoic acid	$C_{18}H_{34}O_3$	298.2508	M+K	337.214	-4	Lipidmaps
[FA methyl,oxo(17:0)] 16-methyl-10-oxo-heptadecanoic acid	$C_{18}H_{34}O_3$	298.2508	M+K	337.214	-4	Lipidmaps
[FA methyl,oxo(17:0)] 2-methyl-4-oxo-heptadecanoic acid	$C_{18}H_{34}O_3$	298.2508	M+K	337.214	-4	Lipidmaps
[FA oxo(18:0)] 11-oxo-octadecanoic acid	$C_{18}H_{34}O_3$	298.2508	M+K	337.214	-4	Lipidmaps
[FA oxo(18:0)] 12-oxo-octadecanoic acid	$C_{18}H_{34}O_3$	298.2508	M+K	337.214	-4	Lipidmaps
[FA oxo(18:0)] 13-oxo-octadecanoic acid	$C_{18}H_{34}O_3$	298.2508	M+K	337.214	-4	Lipidmaps
[FA oxo(18:0)] 14-oxo-octadecanoic acid	$C_{18}H_{34}O_3$	298.2508	M+K	337.214	-4	Lipidmaps
[FA oxo(18:0)] 15-oxo-octadecanoic acid	$C_{18}H_{34}O_3$	298.2508	M+K	337.214	-4	Lipidmaps
[FA oxo(18:0)] 16-oxo-octadecanoic acid	$C_{18}H_{34}O_3$	298.2508	M+K	337.214	-4	Lipidmaps
[FA oxo(18:0)] 17-oxo-octadecanoic acid	$C_{18}H_{34}O_3$	298.2508	M+K	337.214	-4	Lipidmaps
[FA oxo(18:0)] 8-oxo-octadecanoic acid	$C_{18}H_{34}O_3$	298.2508	M+K	337.214	-4	Lipidmaps
[FA (18:0)] 9S,10R-epoxy-octadecanoic acid	$C_{18}H_{34}O_3$	298.2508	M+K	337.214	-4	Lipidmaps
[FA (18:0)] 9R,10S-epoxy-octadecanoic acid	$C_{18}H_{34}O_3$	298.2508	M+K	337.214	-4	Lipidmaps
2R-hydroxy-oleic acid	$C_{18}H_{34}O_3$	298.2508	M+K	337.214	-4	Lipidmaps

MS search for 363.1263 *m/z*

**Compound**	**Formula**	**Compound MW**	**Adduct type**	**Adduct *m/z* calc**	**Delta [ppm]**	**Database**
[FA (16:1)] 7-hexadecenoic acid	$C_{16}H_{30}O_2$	254.2246	M+Ag109	363.1288	-6.8	Lipidmaps
[FA (16:1)] 10Z-hexadecenoic acid	$C_{16}H_{30}O_2$	254.2246	M+Ag109	363.1288	-6.8	Lipidmaps
[FA (16:1)] 10-hexadecenoic acid	$C_{16}H_{30}O_2$	254.2246	M+Ag109	363.1288	-6.8	Lipidmaps
[FA (16:1)] 11-hexadecenoic acid	$C_{16}H_{30}O_2$	254.2246	M+Ag109	363.1288	-6.8	Lipidmaps
[FA (16:1)] 11Z-hexadecenoic acid	$C_{16}H_{30}O_2$	254.2246	M+Ag109	363.1288	-6.8	Lipidmaps
[FA (16:1)] 13-hexadecenoic acid	$C_{16}H_{30}O_2$	254.2246	M+Ag109	363.1288	-6.8	Lipidmaps
[FA (16:1)] 13Z-hexadecenoic acid	$C_{16}H_{30}O_2$	254.2246	M+Ag109	363.1288	-6.8	Lipidmaps
[FA (16:1)] 2Z-hexadecenoic acid	$C_{16}H_{30}O_2$	254.2246	M+Ag109	363.1288	-6.8	Lipidmaps
[FA (16:1)] 3E-hexadecenoic acid	$C_{16}H_{30}O_2$	254.2246	M+Ag109	363.1288	-6.8	Lipidmaps
[FA (16:1)] 6Z-hexadecenoic acid	$C_{16}H_{30}O_2$	254.2246	M+Ag109	363.1288	-6.8	Lipidmaps
Hexadec-7Z-enoic acid	$C_{16}H_{30}O_2$	254.2246	M+Ag109	363.1288	-6.8	Lipidmaps

10E-Tetradecenyl acetate	$C_{16}H_{30}O_2$	254.2246	M+Ag109	363.1288	-6.8	Lipidmaps
11E-Tetradecenyl acetate	$C_{16}H_{30}O_2$	254.2246	M+Ag109	363.1288	-6.8	Lipidmaps
12E-Tetradecenyl acetate	$C_{16}H_{30}O_2$	254.2246	M+Ag109	363.1288	-6.8	Lipidmaps
3E-Tetradecenyl acetate	$C_{16}H_{30}O_2$	254.2246	M+Ag109	363.1288	-6.8	Lipidmaps
5E-Tetradecenyl acetate	$C_{16}H_{30}O_2$	254.2246	M+Ag109	363.1288	-6.8	Lipidmaps
6E-Tetradecenyl acetate	$C_{16}H_{30}O_2$	254.2246	M+Ag109	363.1288	-6.8	Lipidmaps
7E-Tetradecenyl acetate	$C_{16}H_{30}O_2$	254.2246	M+Ag109	363.1288	-6.8	Lipidmaps
8E-Tetradecenyl acetate	$C_{16}H_{30}O_2$	254.2246	M+Ag109	363.1288	-6.8	Lipidmaps
9E-Tetradecenyl acetate	$C_{16}H_{30}O_2$	254.2246	M+Ag109	363.1288	-6.8	Lipidmaps
10Z-Tetradecenyl acetate	$C_{16}H_{30}O_2$	254.2246	M+Ag109	363.1288	-6.8	Lipidmaps
11Z-Tetradecenyl acetate	$C_{16}H_{30}O_2$	254.2246	M+Ag109	363.1288	-6.8	Lipidmaps
12Z-Tetradecenyl acetate	$C_{16}H_{30}O_2$	254.2246	M+Ag109	363.1288	-6.8	Lipidmaps
3Z-Tetradecenyl acetate	$C_{16}H_{30}O_2$	254.2246	M+Ag109	363.1288	-6.8	Lipidmaps
5Z-Tetradecenyl acetate	$C_{16}H_{30}O_2$	254.2246	M+Ag109	363.1288	-6.8	Lipidmaps
6Z-Tetradecenyl acetate	$C_{16}H_{30}O_2$	254.2246	M+Ag109	363.1288	-6.8	Lipidmaps
7Z-Tetradecenyl acetate	$C_{16}H_{30}O_2$	254.2246	M+Ag109	363.1288	-6.8	Lipidmaps
8Z-Tetradecenyl acetate	$C_{16}H_{30}O_2$	254.2246	M+Ag109	363.1288	-6.8	Lipidmaps
9Z-Tetradecenyl acetate	$C_{16}H_{30}O_2$	254.2246	M+Ag109	363.1288	-6.8	Lipidmaps
Ethyl 7E-tetradecenoate	$C_{16}H_{30}O_2$	254.2246	M+Ag109	363.1288	-6.8	Lipidmaps
Ethyl 9E-tetradecenoate	$C_{16}H_{30}O_2$	254.2246	M+Ag109	363.1288	-6.8	Lipidmaps
Ethyl 9Z-tetradecenoate	$C_{16}H_{30}O_2$	254.2246	M+Ag109	363.1288	-6.8	Lipidmaps
Dodecyl 2E-butenoate	$C_{16}H_{30}O_2$	254.2246	M+Ag109	363.1288	-6.8	Lipidmaps
(Z)-7-Dodecenyl butyrate	$C_{16}H_{30}O_2$	254.2246	M+Ag109	363.1288	-6.8	Lipidmaps
15R-Hexadecanolide	$C_{16}H_{30}O_2$	254.2246	M+Ag109	363.1288	-6.8	Lipidmaps
16-Hexadecanolide	$C_{16}H_{30}O_2$	254.2246	M+Ag109	363.1288	-6.8	Lipidmaps
Delta-hexadecalactone	$C_{16}H_{30}O_2$	254.2246	M+Ag109	363.1288	-6.8	Lipidmaps

MS search for 364.1466 *m/z*

Compound	Formula	Compound MW	Adduct type	Adduct *m/z* calc	Delta [ppm]	Database
[PC] 2-valeryl-sn-glycero-3-phosphocholine	$C_{13}H_{28}NO_7P$	341.1603	M+Na	364.1496	-8.1	Lipidmaps

MS search for 365.1445 *m/z*

Compound	Formula	Compound MW	Adduct type	Adduct *m/z* calc	Delta [ppm]	Database
[FA (15:0)] 14-methy-pentadecanoic acid	$C_{16}H_{32}O_2$	256.2402	M+Ag109	365.1444	0.2	Lipidmaps
[FA dimethyl(14:0)] 2,6-dimethyl-tetradecanoic acid	$C_{16}H_{32}O_2$	256.2402	M+Ag109	365.1444	0.2	Lipidmaps
[FA dimethyl(14:0)] 2,8-dimethyl-tetradecanoic acid	$C_{16}H_{32}O_2$	256.2402	M+Ag109	365.1444	0.2	Lipidmaps
[FA methyl(15:0)] 3-methyl-pentadecanoic acid	$C_{16}H_{32}O_2$	256.2402	M+Ag109	365.1444	0.2	Lipidmaps
[FA (3:2/13:0)] 2-propyl-tridecanoic acid	$C_{16}H_{32}O_2$	256.2402	M+Ag109	365.1444	0.2	Lipidmaps
[FA (6:2/10:0)] 2-hexyl-decanoic acid	$C_{16}H_{32}O_2$	256.2402	M+Ag109	365.1444	0.2	Lipidmaps
[FA ethyl,methyl(13:0)] 3-ethyl-3-methyl-tridecanoic acid	$C_{16}H_{32}O_2$	256.2402	M+Ag109	365.1444	0.2	Lipidmaps
[FA (7:2/9:0)] 2-heptyl-nonanoic acid	$C_{16}H_{32}O_2$	256.2402	M+Ag109	365.1444	0.2	Lipidmaps
[FA ethyl(14:0)] 6-ethyl-tetradecanoic acid	$C_{16}H_{32}O_2$	256.2402	M+Ag109	365.1444	0.2	Lipidmaps
[FA dimethyl(14:0)] 2,4-dimethyl-tetradecanoic acid	$C_{16}H_{32}O_2$	256.2402	M+Ag109	365.1444	0.2	Lipidmaps
[FA dimethyl(14:0)] 3,5-dimethyl-tetradecanoic acid	$C_{16}H_{32}O_2$	256.2402	M+Ag109	365.1444	0.2	Lipidmaps
[FA (6:2/10:0)] 4-hexyl-decanoic acid	$C_{16}H_{32}O_2$	256.2402	M+Ag109	365.1444	0.2	Lipidmaps
[FA ethyl(4:2/10:0)] 2-ethyl-2-butyl-decanoic acid	$C_{16}H_{32}O_2$	256.2402	M+Ag109	365.1444	0.2	Lipidmaps
[FA methyl(15:0)] 13-methyl-pentadecanoic acid	$C_{16}H_{32}O_2$	256.2402	M+Ag109	365.1444	0.2	Lipidmaps
13,13-dimethyl-tetradecanoic acid	$C_{16}H_{32}O_2$	256.2402	M+Ag109	365.1444	0.2	Lipidmaps
14:0(10Me,13Me)	$C_{16}H_{32}O_2$	256.2402	M+Ag109	365.1444	0.2	Lipidmaps
2-hydroxyhexadecanal	$C_{16}H_{32}O_2$	256.2402	M+Ag109	365.1444	0.2	Lipidmaps
Tetradecyl acetate	$C_{16}H_{32}O_2$	256.2402	M+Ag109	365.1444	0.2	Lipidmaps
Dodecyl butyrate	$C_{16}H_{32}O_2$	256.2402	M+Ag109	365.1444	0.2	Lipidmaps
Decyl hexanoate	$C_{16}H_{32}O_2$	256.2402	M+Ag109	365.1444	0.2	Lipidmaps
Octyl octanoate	$C_{16}H_{32}O_2$	256.2402	M+Ag109	365.1444	0.2	Lipidmaps
Hexyl decanoate	$C_{16}H_{32}O_2$	256.2402	M+Ag109	365.1444	0.2	Lipidmaps

Ethyl tetradecanoate	$C_{16}H_{32}O_2$	256.2402	M+Ag10 9	365.1444	0.2	Lipidmaps
Formyl 2,6,10-trimethyl-dodecanoate	$C_{16}H_{32}O_2$	256.2402	M+Ag10 9	365.1444	0.2	Lipidmaps
7-Methyloctyl 5-methylhexanoate	$C_{16}H_{32}O_2$	256.2402	M+Ag10 9	365.1444	0.2	Lipidmaps

MS search for 392.1792 *m/z*

**Compound**	**Formula**	**Compound MW**	**Adduct type**	**Adduct *m/z* calc**	**Delta [ppm]**	**Database**
PC(7:0/0:0)	$C_{15}H_{32}NO_7P$	369.1916	M+Na	392.1809	-4.2	Lipidmaps
[PC (7:0)] 1-heptanoyl-sn-glycero-3-phosphocholine	$C_{15}H_{32}NO_7P$	369.1916	M+Na	392.1809	-4.2	Lipidmaps
PC(0:0/7:0)	$C_{15}H_{32}NO_7P$	369.1916	M+Na	392.1809	-4.2	Lipidmaps
[PC (7:0)] 2-heptanoyl-sn-glycero-3-phosphocholine	$C_{15}H_{32}NO_7P$	369.1916	M+Na	392.1809	-4.2	Lipidmaps

MS search for 419.2934 *m/z*

**Compound**	**Formula**	**Compound MW**	**Adduct type**	**Adduct *m/z* calc**	**Delta [ppm]**	**Database**
[FA oxo(24:0)] 15-oxo-18Z-tetracosenoic acid	$C_{24}H_{44}O_3$	380.329	M+K	419.2922	2.8	Lipidmaps
[ST (5:0/2:0)] (5Z,7E,23E)-(3S)-9,10-seco-5,7,10(19),16,23-cholestapentaene-3,25-diol	$C_{27}H_{40}O_2$	396.3028	M+Na	419.292	3.2	Lipidmaps
[ST (5:0/2:0)] (5Z,7E,23Z)-(3S)-9,10-seco-5,7,10(19),16,23-cholestapentaene-3,25-diol	$C_{27}H_{40}O_2$	396.3028	M+Na	419.292	3.2	Lipidmaps
[ST (2:0/2:0/2:0)] (5Z,7E)-(3S)-9,10-seco-5,7,10(19)-cholestatrien-23-yne-3,25-diol	$C_{27}H_{40}O_2$	396.3028	M+Na	419.292	3.2	Lipidmaps
[ST hydroxy(4:0)] (22E)-(8S)-3-hydroxy-9,10-seco-1,3,5(10),22-cholestatetraen-9-one	$C_{27}H_{40}O_2$	396.3028	M+Na	419.292	3.2	Lipidmaps

MS search for 447.2651 *m/z*

**Compound**	**Formula**	**Compound MW**	**Adduct type**	**Adduct *m/z* calc**	**Delta [ppm]**	**Database**
4Z,7Z,10Z,13Z,16Z,19Z,22Z,25Z-octacosaoctaenoic acid	$C_{28}H_{40}O_2$	408.3028	M+K	447.266	-2	Lipidmaps

MS search for 449.2477 *m/z*

**Compound**	**Formula**	**Compound MW**	**Adduct type**	**Adduct *m/z* calc**	**Delta [ppm]**	**Database**
[ST hydroxy(4:0)] (5Z,7E,22E)-(1S,3R)-1,3-dihydroxy-26,27-cyclo-9,10-seco-5,7,10(19),22-cholestatetraen-24-one	$C_{27}H_{38}O_3$	410.2821	M+K	449.2453	5.4	Lipidmaps

[ST (2:0/2:0/2:0)] (5Z,7E)-(1S,3R)-24,25-epoxy-9,10-seco-5,7,10(19)-cholestatrien-22-yne-1,3-diol	$C_{27}H_{38}O_3$	410.2821	M+K	449.2453	5.4	Lipidmaps
[ST (2:0/2:0/2:0)] (5Z,7E)-(1S,3R)-25,26-epoxy-9,10-seco-5,7,10(19)-cholestatrien-23-yne-1,3-diol	$C_{27}H_{38}O_3$	410.2821	M+K	449.2453	5.4	Lipidmaps
[ST (2:0/2:0/2:0)] (5Z,7E)-(1S,3R,20S)-25,26-epoxy-9,10-seco-5,7,10(19)-cholestatrien-23-yne-1,3-diol	$C_{27}H_{38}O_3$	410.2821	M+K	449.2453	5.4	Lipidmaps
[ST (4:0/3:0)] (5Z,7E)-(1S,3R)-9,10-seco-5,7,10(19),16-cholestatetraen-23-yne-1,3,25-triol	$C_{27}H_{38}O_3$	410.2821	M+K	449.2453	5.4	Lipidmaps
[FA hydroxy,oxo(2:0)] 9S,15S-dihydroxy-11-oxo-5Z,13E-prostadienoic acid 2-glyceryl ester	$C_{23}H_{38}O_7$	426.2618	M+Na	449.251	-7.3	Lipidmaps
[FA hydroxy,oxo(2:0)] 9S,15S-dihydroxy-11-oxo-5Z,13E-prostadienoic acid 1(3)-glyceryl ester	$C_{23}H_{38}O_7$	426.2618	M+Na	449.251	-7.3	Lipidmaps
[FA oxo,hydroxy(2:0)] 9-oxo-11R,15S-dihydroxy-5Z,13E-prostadienoic acid 2-glyceryl ester	$C_{23}H_{38}O_7$	426.2618	M+Na	449.251	-7.3	Lipidmaps
[FA oxo,hydroxy(2:0)] 9-oxo-11R,15S-dihydroxy-5Z,13E-prostadienoic acid 1(3)-glyceryl ester	$C_{23}H_{38}O_7$	426.2618	M+Na	449.251	-7.3	Lipidmaps
[FA hydroxy(2:0)] 9S,11R-epidioxy-15S-hydroxy-5Z,13E-prostadienoic acid 2-glyceryl ester	$C_{23}H_{38}O_7$	426.2618	M+Na	449.251	-7.3	Lipidmaps
[FA hydroxy(2:0)] 9S,11R-epidioxy-15S-hydroxy-5Z,13E-prostadienoic acid 1(3)-glyceryl ester	$C_{23}H_{38}O_7$	426.2618	M+Na	449.251	-7.3	Lipidmaps

MS search for 453.2299 *m/z*

**Compound**	**Formula**	**Compound MW**	**Adduct type**	**Adduct *m/z* calc**	**Delta [ppm]**	**Database**
[Fv] Kushenol D	$C_{27}H_{32}O_6$	452.2199	M+H	453.2272	6	Lipidmaps
[Fv hydroxy,dimethoxy(9:1)] (2S)-7,4'-Dihydroxy-8-lavandulyl-5,2'-dimethoxyflavanone	$C_{27}H_{32}O_6$	452.2199	M+H	453.2272	6	Lipidmaps
[Fv] Amoradinin	$C_{27}H_{32}O_6$	452.2199	M+H	453.2272	6	Lipidmaps

MS search for 478.2714 *m/z*

**Compound**	**Formula**	**Compound MW**	**Adduct type**	**Adduct *m/z* calc**	**Delta [ppm]**	**Database**

PC(O-12:0/O-1:0)	$C_{21}H_{46}NO_6P$	439.3063	M+K	478.2694	4.1	Lipidmaps
[PC methyl(12:2)] 1-dodecyl-2-methyl-sn-glycero-3-phosphocholine	$C_{21}H_{46}NO_6P$	439.3063	M+K	478.2694	4.1	Lipidmaps
[PE (16:2)] 1-hexadecyl-sn-glycero-3-phosphoethanolamine	$C_{21}H_{46}NO_6P$	439.3063	M+K	478.2694	4.1	Lipidmaps

MS search for 503.282 *m*/*z*

**Compound**	**Formula**	**Compound MW**	**Adduct type**	**Adduct *m*/*z* calc**	**Delta [ppm]**	**Database**
[FA (26:0)] 9-hexacosenoic acid	$C_{26}H_{50}O_2$	394.3811	M+Ag109	503.2853	-6.5	Lipidmaps
[FA (26:0)] 17Z-hexacosenoic acid	$C_{26}H_{50}O_2$	394.3811	M+Ag109	503.2853	-6.5	Lipidmaps
[FA (26:0)] 17-hexacosenoic acid	$C_{26}H_{50}O_2$	394.3811	M+Ag109	503.2853	-6.5	Lipidmaps
[FA (26:0)] 9Z-hexacosenoic acid	$C_{26}H_{50}O_2$	394.3811	M+Ag109	503.2853	-6.5	Lipidmaps
Lauryl myristoleate	$C_{26}H_{50}O_2$	394.3811	M+Ag109	503.2853	-6.5	Lipidmaps
Myristoleyl laurate	$C_{26}H_{50}O_2$	394.3811	M+Ag109	503.2853	-6.5	Lipidmaps
4-Methyl-3-heptyl oleate	$C_{26}H_{50}O_2$	394.3811	M+Ag109	503.2853	-6.5	Lipidmaps
26-Hexacosanolide	$C_{26}H_{50}O_2$	394.3811	M+Ag109	503.2853	-6.5	Lipidmaps
22:1(5Z)(9Me,13Me,17Me,21Me)	$C_{26}H_{50}O_2$	394.3811	M+Ag109	503.2853	-6.5	Lipidmaps

MS search for 559.3414 *m*/*z*

**Compound**	**Formula**	**Compound MW**	**Adduct type**	**Adduct *m*/*z* calc**	**Delta [ppm]**	**Database**
PA(12:0/12:0)	$C_{27}H_{53}O_8P$	536.3478	M+Na	559.337	7.8	Lipidmaps

MS search for 569.3915 *m*/*z*

**Compound**	**Formula**	**Compound MW**	**Adduct type**	**Adduct *m*/*z* calc**	**Delta [ppm]**	**Database**
3-Hexaprenyl-4-hydroxybenzoic acid	$C_{37}H_{54}O_3$	546.4073	M+Na	569.3965	-8.8	Lipidmaps

MS search for 608.4593 *m*/*z*

**Compound**	**Formula**	**Compound MW**	**Adduct type**	**Adduct *m*/*z* calc**	**Delta [ppm]**	**Database**
[PC (24:0)] 1-tetracosanoyl-sn-glycero-3-phosphocholine	$C_{32}H_{66}NO_7P$	607.4577	M+H	608.465	-9.3	Lipidmaps

MS search for 615.4225 *m*/*z*

Compound	Formula	Compound MW	Adduct type	Adduct *m/z* calc	Delta [ppm]	Database
[FA (26:0/2:0)] 1-(O-alpha-D-glucopyranosyl)-(3R,25R)-hexacosanediol	$C_{32}H_{64}O_8$	576.4601	M+K	615.4233	-1.3	Lipidmaps
[FA (26:0/2:0)] 1-(O-alpha-D-glucopyranosyl)-(3S,25R)-hexacosanediol	$C_{32}H_{64}O_8$	576.4601	M+K	615.4233	-1.3	Lipidmaps

MS search for 643.4271 *m/z*

Compound	Formula	Compound MW	Adduct type	Adduct *m/z* calc	Delta [ppm]	Database
PA(18:0/12:0)	$C_{33}H_{65}O_8P$	620.4417	M+Na	643.4309	-6	Lipidmaps
PA(17:0/13:0)	$C_{33}H_{65}O_8P$	620.4417	M+Na	643.4309	-6	Lipidmaps
PA(13:0/17:0)	$C_{33}H_{65}O_8P$	620.4417	M+Na	643.4309	-6	Lipidmaps
PA(12:0/18:0)	$C_{33}H_{65}O_8P$	620.4417	M+Na	643.4309	-6	Lipidmaps
PA(16:0/14:0)	$C_{33}H_{65}O_8P$	620.4417	M+Na	643.4309	-6	Lipidmaps
PA(15:0/15:0)	$C_{33}H_{65}O_8P$	620.4417	M+Na	643.4309	-6	Lipidmaps
PA(14:0/16:0)	$C_{33}H_{65}O_8P$	620.4417	M+Na	643.4309	-6	Lipidmaps
PA(30:0)	$C_{33}H_{65}O_8P$	620.4417	M+Na	643.4309	-6	
PA(12:0/20:3(8Z,11Z,14Z))	$C_{35}H_{63}O_8P$	642.4261	M+H	643.4333	-9.7	Lipidmaps
PA(14:0/18:3(6Z,9Z,12Z))	$C_{35}H_{63}O_8P$	642.4261	M+H	643.4333	-9.7	Lipidmaps
PA(14:1(9Z)/18:2(9Z,12Z))	$C_{35}H_{63}O_8P$	642.4261	M+H	643.4333	-9.7	Lipidmaps
PA(15:1(9Z)/17:2(9Z,12Z))	$C_{35}H_{63}O_8P$	642.4261	M+H	643.4333	-9.7	Lipidmaps
PA(17:2(9Z,12Z)/15:1(9Z))	$C_{35}H_{63}O_8P$	642.4261	M+H	643.4333	-9.7	Lipidmaps
PA(18:2(9Z,12Z)/14:1(9Z))	$C_{35}H_{63}O_8P$	642.4261	M+H	643.4333	-9.7	Lipidmaps
PA(18:3(6Z,9Z,12Z)/14:0)	$C_{35}H_{63}O_8P$	642.4261	M+H	643.4333	-9.7	Lipidmaps
PA(18:3(9Z,12Z,15Z)/14:0)	$C_{35}H_{63}O_8P$	642.4261	M+H	643.4333	-9.7	Lipidmaps
PA(20:3(8Z,11Z,14Z)/12:0)	$C_{35}H_{63}O_8P$	642.4261	M+H	643.4333	-9.7	Lipidmaps
PA(14:0/18:3(9Z,12Z,15Z))	$C_{35}H_{63}O_8P$	642.4261	M+H	643.4333	-9.7	Lipidmaps
PA(32:3)	$C_{35}H_{63}O_8P$	642.4261	M+H	643.4333	-9.7	Lipidmaps

MS search for 645.5618 *m/z*

Compound	Formula	Compound MW	Adduct type	Adduct *m/z* calc	Delta [ppm]	Database
Stigmast-5,22E-dien-3beta-yl (7Z,10Z,13Z-hexadecatrienoate)	$C_{45}H_{72}O_2$	644.5532	M+H	645.5605	2	Lipidmaps
[ST (16:0)] (5Z,7E)-(3S)-9,10-seco-5,7,10(19)-cholestatriene-3-yl hexadecanoate	$C_{43}H_{74}O_2$	622.5689	M+Na	645.5581	5.7	Lipidmaps
Cholest-5-en-3β-yl (7Z-hexadecenoate)	$C_{43}H_{74}O_2$	622.5689	M+Na	645.5581	5.7	Lipidmaps

MS search for 675.5169 *m/z*

Compound	Formula	Compound MW	Adduct type	Adduct *m/z* calc	Delta [ppm	Database

					]	
[GL (8:0/8:0)] 1-(8-[3]-ladderane-octanyl)-2-(8-[3]-ladderane-octanyl)-sn-glycerol	$C_{43}H_{72}O_3$	636.5482	M+K	675.5113	8.3	Lipidmaps

MS search for 688.3903 ***m/z***

Compound	Formula	Compound MW	Adduct type	Adduct *m/z* calc	Delta [ppm]	Database
PC(16:0/9:0(CHO))	$C_{33}H_{64}NO_9P$	649.4319	M+K	688.395	-6.9	Lipidmaps

MS search for 699.4998 ***m/z***

Compound	Formula	Compound MW	Adduct type	Adduct *m/z* calc	Delta [ppm]	Database
PA(14:1(9Z)/22:2(13Z,16Z))	$C_{39}H_{71}O_8P$	698.4887	M+H	699.4959	5.5	Lipidmaps
PA(16:1(9Z)/20:2(11Z,14Z))	$C_{39}H_{71}O_8P$	698.4887	M+H	699.4959	5.5	Lipidmaps
PA(17:2(9Z,12Z)/19:1(9Z))	$C_{39}H_{71}O_8P$	698.4887	M+H	699.4959	5.5	Lipidmaps
PA(18:0/18:3(6Z,9Z,12Z))	$C_{39}H_{71}O_8P$	698.4887	M+H	699.4959	5.5	Lipidmaps
PA(18:0/18:3(9Z,12Z,15Z))	$C_{39}H_{71}O_8P$	698.4887	M+H	699.4959	5.5	Lipidmaps
PA(18:2(9Z,12Z)/18:1(9Z))	$C_{39}H_{71}O_8P$	698.4887	M+H	699.4959	5.5	Lipidmaps
PA(18:3(6Z,9Z,12Z)/18:0)	$C_{39}H_{71}O_8P$	698.4887	M+H	699.4959	5.5	Lipidmaps
PA(18:3(9Z,12Z,15Z)/18:0)	$C_{39}H_{71}O_8P$	698.4887	M+H	699.4959	5.5	Lipidmaps
PA(19:1(9Z)/17:2(9Z,12Z))	$C_{39}H_{71}O_8P$	698.4887	M+H	699.4959	5.5	Lipidmaps
PA(20:2(11Z,14Z)/16:1(9Z))	$C_{39}H_{71}O_8P$	698.4887	M+H	699.4959	5.5	Lipidmaps
PA(20:3(8Z,11Z,14Z)/16:0)	$C_{39}H_{71}O_8P$	698.4887	M+H	699.4959	5.5	Lipidmaps
PA(22:2(13Z,16Z)/14:1(9Z))	$C_{39}H_{71}O_8P$	698.4887	M+H	699.4959	5.5	Lipidmaps
PA(16:0/20:3(8Z,11Z,14Z))	$C_{39}H_{71}O_8P$	698.4887	M+H	699.4959	5.5	Lipidmaps
PA(18:1(9Z)/18:2(9Z,12Z))	$C_{39}H_{71}O_8P$	698.4887	M+H	699.4959	5.5	Lipidmaps
PA(36:3)	$C_{39}H_{71}O_8P$	698.4887	M+H	699.4959	5.5	Lipidmaps
Tetracosanyl palmitoleate	$C_{40}H_{78}O_2$	590.6002	M+Ag109	699.5044	-6.5	Lipidmaps

MS search for 701.5125 ***m/z***

Compound	Formula	Compound MW	Adduct type	Adduct *m/z* calc	Delta [ppm]	Database
PA(14:0/22:2(13Z,16Z))	$C_{39}H_{73}O_8P$	700.5043	M+H	701.5116	1.3	Lipidmaps
PA(14:1(9Z)/22:1(11Z))	$C_{39}H_{73}O_8P$	700.5043	M+H	701.5116	1.3	Lipidmaps
PA(16:0/20:2(11Z,14Z))	$C_{39}H_{73}O_8P$	700.5043	M+H	701.5116	1.3	Lipidmaps
PA(16:1(9Z)/20:1(11Z))	$C_{39}H_{73}O_8P$	700.5043	M+H	701.5116	1.3	Lipidmaps
PA(17:1(9Z)/19:1(9Z))	$C_{39}H_{73}O_8P$	700.5043	M+H	701.5116	1.3	Lipidmaps
PA(17:2(9Z,12Z)/19:0)	$C_{39}H_{73}O_8P$	700.5043	M+H	701.5116	1.3	Lipidmaps
PA(19:0/17:2(9Z,12Z))	$C_{39}H_{73}O_8P$	700.5043	M+H	701.5116	1.3	Lipidmaps
PA(19:1(9Z)/17:1(9Z))	$C_{39}H_{73}O_8P$	700.5043	M+H	701.5116	1.3	Lipidmaps
PA(20:1(11Z)/16:1(9Z))	$C_{39}H_{73}O_8P$	700.5043	M+H	701.5116	1.3	Lipidmaps
PA(20:2(11Z,14Z)/16:0)	$C_{39}H_{73}O_8P$	700.5043	M+H	701.5116	1.3	Lipidmaps

PA(22:1(11Z)/14:1(9Z))	$C_{39}H_{73}O_8P$	700.5043	M+H	701.5116	1.3	Lipidmaps
PA(22:2(13Z,16Z)/14:0)	$C_{39}H_{73}O_8P$	700.5043	M+H	701.5116	1.3	Lipidmaps
PA(18:2(9Z,12Z)/18:0)	$C_{39}H_{73}O_8P$	700.5043	M+H	701.5116	1.3	Lipidmaps
PA(36:2)	$C_{39}H_{73}O_8P$	700.5043	M+H	701.5116	1.3	Lipidmaps

MS search for 717.5011 *m/z*

**Compound**	**Formula**	**Compound MW**	**Adduct type**	**Adduct *m/z* calc**	**Delta [ppm]**	**Database**
PG(O-16:0/15:0)	$C_{37}H_{75}O_9P$	694.5149	M+Na	717.5041	-4.2	Lipidmaps
PG(O-18:0/13:0)	$C_{37}H_{75}O_9P$	694.5149	M+Na	717.5041	-4.2	Lipidmaps

MS search for 727.5254 *m/z*

**Compound**	**Formula**	**Compound MW**	**Adduct type**	**Adduct *m/z* calc**	**Delta [ppm]**	**Database**
PA(17:0/19:0)	$C_{39}H_{77}O_8P$	704.5356	M+Na	727.5248	0.8	Lipidmaps
PA(19:0/17:0)	$C_{39}H_{77}O_8P$	704.5356	M+Na	727.5248	0.8	Lipidmaps
PA(21:0/15:0)	$C_{39}H_{77}O_8P$	704.5356	M+Na	727.5248	0.8	Lipidmaps
PA(22:0/14:0)	$C_{39}H_{77}O_8P$	704.5356	M+Na	727.5248	0.8	Lipidmaps
PA(20:0/16:0)	$C_{39}H_{77}O_8P$	704.5356	M+Na	727.5248	0.8	Lipidmaps
PA(15:0/21:0)	$C_{39}H_{77}O_8P$	704.5356	M+Na	727.5248	0.8	Lipidmaps
PA(14:0/22:0)	$C_{39}H_{77}O_8P$	704.5356	M+Na	727.5248	0.8	Lipidmaps
PA(16:0/20:0)	$C_{39}H_{77}O_8P$	704.5356	M+Na	727.5248	0.8	Lipidmaps
PA(36:0)	$C_{39}H_{77}O_8P$	704.5356	M+Na	727.5248	0.8	Lipidmaps

MS search for 731.489 *m/z*

**Compound**	**Formula**	**Compound MW**	**Adduct type**	**Adduct *m/z* calc**	**Delta [ppm]**	**Database**
PG(13:0/20:3(8Z,11Z,14Z))	$C_{39}H_{71}O_{10}P$	730.4785	M+H	731.4858	4.4	Lipidmaps
PG(15:0/18:3(6Z,9Z,12Z))	$C_{39}H_{71}O_{10}P$	730.4785	M+H	731.4858	4.4	Lipidmaps
PG(15:0/18:3(9Z,12Z,15Z))	$C_{39}H_{71}O_{10}P$	730.4785	M+H	731.4858	4.4	Lipidmaps
PG(15:1(9Z)/18:2(9Z,12Z))	$C_{39}H_{71}O_{10}P$	730.4785	M+H	731.4858	4.4	Lipidmaps
PG(16:1(9Z)/17:2(9Z,12Z))	$C_{39}H_{71}O_{10}P$	730.4785	M+H	731.4858	4.4	Lipidmaps
PG(17:2(9Z,12Z)/16:1(9Z))	$C_{39}H_{71}O_{10}P$	730.4785	M+H	731.4858	4.4	Lipidmaps
PG(18:2(9Z,12Z)/15:1(9Z))	$C_{39}H_{71}O_{10}P$	730.4785	M+H	731.4858	4.4	Lipidmaps
PG(18:3(6Z,9Z,12Z)/15:0)	$C_{39}H_{71}O_{10}P$	730.4785	M+H	731.4858	4.4	Lipidmaps
PG(18:3(9Z,12Z,15Z)/15:0)	$C_{39}H_{71}O_{10}P$	730.4785	M+H	731.4858	4.4	Lipidmaps
PG(20:3(8Z,11Z,14Z)/13:0)	$C_{39}H_{71}O_{10}P$	730.4785	M+H	731.4858	4.4	Lipidmaps

MS search for 733.502 *m/z*

**Compound**	**Formula**	**Compound MW**	**Adduct type**	**Adduct *m/z* calc**	**Delta [ppm]**	**Database**
PG(13:0/20:2(11Z,14Z))	$C_{39}H_{73}O_{10}P$	732.4941	M+H	733.5014	0.8	Lipidmaps
PG(14:1(9Z)/19:1(9Z))	$C_{39}H_{73}O_{10}P$	732.4941	M+H	733.5014	0.8	Lipidmaps

PG(15:1(9Z)/18:1(9Z))	$C_{39}H_{73}O_{10}P$	732.4941	M+H	733.5014	0.8	Lipidmaps
PG(16:0/17:2(9Z,12Z))	$C_{39}H_{73}O_{10}P$	732.4941	M+H	733.5014	0.8	Lipidmaps
PG(16:1(9Z)/17:1(9Z))	$C_{39}H_{73}O_{10}P$	732.4941	M+H	733.5014	0.8	Lipidmaps
PG(17:1(9Z)/16:1(9Z))	$C_{39}H_{73}O_{10}P$	732.4941	M+H	733.5014	0.8	Lipidmaps
PG(17:2(9Z,12Z)/16:0)	$C_{39}H_{73}O_{10}P$	732.4941	M+H	733.5014	0.8	Lipidmaps
PG(18:1(9Z)/15:1(9Z))	$C_{39}H_{73}O_{10}P$	732.4941	M+H	733.5014	0.8	Lipidmaps
PG(18:2(9Z,12Z)/15:0)	$C_{39}H_{73}O_{10}P$	732.4941	M+H	733.5014	0.8	Lipidmaps
PG(19:1(9Z)/14:1(9Z))	$C_{39}H_{73}O_{10}P$	732.4941	M+H	733.5014	0.8	Lipidmaps
PG(20:2(11Z,14Z)/13:0)	$C_{39}H_{73}O_{10}P$	732.4941	M+H	733.5014	0.8	Lipidmaps
PG(15:0/18:2(9Z,12Z))	$C_{39}H_{73}O_{10}P$	732.4941	M+H	733.5014	0.8	Lipidmaps

MS search for 753.1973 *m/z*

**Compound**	**Formula**	**Compound MW**	**Adduct type**	**Adduct *m/z* calc**	**Delta [ppm]**	**Database**
PI(22:6(4Z,7Z,10Z,13Z,16Z,19Z)/0:0)	$C_{31}H_{49}O_{12}P$	644.2962	M+Ag109	753.2004	-4.1	Lipidmaps

MS search for 753.3986 *m/z*

**Compound**	**Formula**	**Compound MW**	**Adduct type**	**Adduct *m/z* calc**	**Delta [ppm]**	**Database**
N-(nonadecanoyl)-4E,6E-tetradecasphingadienine-1-phosphoethanolamine	$C_{35}H_{69}N_2O_6P$	644.4893	M+Ag109	753.3935	6.7	Lipidmaps
N-(octadecanoyl)-4E,6E-pentadecasphingadienine-1-phosphoethanolamine	$C_{35}H_{69}N_2O_6P$	644.4893	M+Ag109	753.3935	6.7	Lipidmaps

MS search for 753.5419 *m/z*

**Compound**	**Formula**	**Compound MW**	**Adduct type**	**Adduct *m/z* calc**	**Delta [ppm]**	**Database**
PA(18:2(9Z,12Z)/22:2(13Z,16Z))	$C_{43}H_{77}O_8P$	752.5356	M+H	753.5429	-1.3	Lipidmaps
PA(18:3(6Z,9Z,12Z)/22:1(11Z))	$C_{43}H_{77}O_8P$	752.5356	M+H	753.5429	-1.3	Lipidmaps
PA(18:3(9Z,12Z,15Z)/22:1(11Z))	$C_{43}H_{77}O_8P$	752.5356	M+H	753.5429	-1.3	Lipidmaps
PA(18:4(6Z,9Z,12Z,15Z)/22:0)	$C_{43}H_{77}O_8P$	752.5356	M+H	753.5429	-1.3	Lipidmaps
PA(20:1(11Z)/20:3(8Z,11Z,14Z))	$C_{43}H_{77}O_8P$	752.5356	M+H	753.5429	-1.3	Lipidmaps
PA(20:2(11Z,14Z)/20:2(11Z,14Z))	$C_{43}H_{77}O_8P$	752.5356	M+H	753.5429	-1.3	Lipidmaps
PA(20:3(8Z,11Z,14Z)/20:1(11Z))	$C_{43}H_{77}O_8P$	752.5356	M+H	753.5429	-1.3	Lipidmaps
PA(20:4(5Z,8Z,11Z,14Z)/20:0)	$C_{43}H_{77}O_8P$	752.5356	M+H	753.5429	-1.3	Lipidmaps
PA(22:0/18:4(6Z,9Z,12Z,15Z))	$C_{43}H_{77}O_8P$	752.5356	M+H	753.5429	-1.3	Lipidmaps
PA(22:1(11Z)/18:3(6Z,9Z,12Z))	$C_{43}H_{77}O_8P$	752.5356	M+H	753.5429	-1.3	Lipidmaps
PA(22:1(11Z)/18:3(9Z,12Z,15Z))	$C_{43}H_{77}O_8P$	752.5356	M+H	753.5429	-1.3	Lipidmaps
PA(22:2(13Z,16Z)/18:2(9Z,12Z))	$C_{43}H_{77}O_8P$	752.5356	M+H	753.5429	-1.3	Lipidmaps
PA(22:4(7Z,10Z,13Z,16Z)/18:0)	$C_{43}H_{77}O_8P$	752.5356	M+H	753.5429	-1.3	Lipidmaps
PA(20:0/20:4(5Z,8Z,11Z,14Z))	$C_{43}H_{77}O_8P$	752.5356	M+H	753.5429	-1.3	Lipidmaps
PA(18:0/22:4(7Z,10Z,13Z,16Z))	$C_{43}H_{77}O_8P$	752.5356	M+H	753.5429	-1.3	Lipidmaps

TG(12:0/12:0/18:4(6Z,9Z,12Z,15Z))[iso3]	$C_{45}H_{78}O_6$	714.5798	M+K	753.543	-1.5	Lipidmaps

MS search for 755.5594 *m/z*

Compound	Formula	Compound MW	Adduct type	Adduct *m/z* calc	Delta [ppm]	Database
TG(14:1(9Z)/14:1(9Z)/14:1(9Z))	$C_{45}H_{80}O_6$	716.5955	M+K	755.5587	1	Lipidmaps
TG(12:0/12:0/18:3(6Z,9Z,12Z))[iso3]	$C_{45}H_{80}O_6$	716.5955	M+K	755.5587	1	Lipidmaps
TG(12:0/12:0/18:3(9Z,12Z,15Z))[iso3]	$C_{45}H_{80}O_6$	716.5955	M+K	755.5587	1	Lipidmaps

MS search for 757.5609 *m/z*

Compound	Formula	Compound MW	Adduct type	Adduct *m/z* calc	Delta [ppm]	Database
SM(d18:0/17:0)	$C_{40}H_{83}N_2O_6P$	718.5989	M+K	757.562	-1.5	Lipidmaps

MS search for 759.522 *m/z*

Compound	Formula	Compound MW	Adduct type	Adduct *m/z* calc	Delta [ppm]	Database
PG(15:0/20:3(8Z,11Z,14Z))	$C_{41}H_{75}O_{10}P$	758.5098	M+H	759.5171	6.5	Lipidmaps
PG(15:1(9Z)/20:2(11Z,14Z))	$C_{41}H_{75}O_{10}P$	758.5098	M+H	759.5171	6.5	Lipidmaps
PG(17:0/18:3(6Z,9Z,12Z))	$C_{41}H_{75}O_{10}P$	758.5098	M+H	759.5171	6.5	Lipidmaps
PG(17:0/18:3(9Z,12Z,15Z))	$C_{41}H_{75}O_{10}P$	758.5098	M+H	759.5171	6.5	Lipidmaps
PG(17:1(9Z)/18:2(9Z,12Z))	$C_{41}H_{75}O_{10}P$	758.5098	M+H	759.5171	6.5	Lipidmaps
PG(17:2(9Z,12Z)/18:1(9Z))	$C_{41}H_{75}O_{10}P$	758.5098	M+H	759.5171	6.5	Lipidmaps
PG(18:1(9Z)/17:2(9Z,12Z))	$C_{41}H_{75}O_{10}P$	758.5098	M+H	759.5171	6.5	Lipidmaps
PG(18:2(9Z,12Z)/17:1(9Z))	$C_{41}H_{75}O_{10}P$	758.5098	M+H	759.5171	6.5	Lipidmaps
PG(18:3(6Z,9Z,12Z)/17:0)	$C_{41}H_{75}O_{10}P$	758.5098	M+H	759.5171	6.5	Lipidmaps
PG(18:3(9Z,12Z,15Z)/17:0)	$C_{41}H_{75}O_{10}P$	758.5098	M+H	759.5171	6.5	Lipidmaps
PG(20:2(11Z,14Z)/15:1(9Z))	$C_{41}H_{75}O_{10}P$	758.5098	M+H	759.5171	6.5	Lipidmaps
PG(20:3(8Z,11Z,14Z)/15:0)	$C_{41}H_{75}O_{10}P$	758.5098	M+H	759.5171	6.5	Lipidmaps

MS search for 783.598 *m/z*

Compound	Formula	Compound MW	Adduct type	Adduct *m/z* calc	Delta [ppm]	Database
MGDG(18:1(9Z)/18:1(9Z))	$C_{45}H_{82}O_{10}$	782.5908	M+H	783.5981	-0.1	Lipidmaps
MGDG(18:0(9Z)/18:2(9Z,12Z))	$C_{45}H_{82}O_{10}$	782.5908	M+H	783.5981	-0.1	Lipidmaps

MS search for 787.6659 *m/z*

Compound	Formula	Compound MW	Adduct type	Adduct *m/z* calc	Delta [ppm]	Database
SM(d16:1/24:0)	$C_{45}H_{91}N_2O_6P$	786.6615	M+H	787.6688	-3.6	Lipidmaps

MS search for 798.3673 *m/z*

Compound	Formula	Compound MW	Adduct type	Adduct *m/z* calc	Delta [ppm]	Database
PS(P-16:0/14:1(9Z))	$C_{36}H_{68}NO_9P$	689.4632	M+Ag109	798.3674	-0.1	Lipidmaps

MS search for 817.4395 *m/z*

Compound	Formula	Compound MW	Adduct type	Adduct *m/z* calc	Delta [ppm]	Database
PG(15:1(9Z)/22:6(4Z,7Z,10Z,13Z,16Z,19Z))	$C_{43}H_{71}O_{10}P$	778.4785	M+K	817.4416	-2.6	Lipidmaps
PG(17:2(9Z,12Z)/20:5(5Z,8Z,11Z,14Z,17Z))	$C_{43}H_{71}O_{10}P$	778.4785	M+K	817.4416	-2.6	Lipidmaps
PG(20:5(5Z,8Z,11Z,14Z,17Z)/17:2(9Z,12Z))	$C_{43}H_{71}O_{10}P$	778.4785	M+K	817.4416	-2.6	Lipidmaps
PG(22:6(4Z,7Z,10Z,13Z,16Z,19Z)/15:1(9Z))	$C_{43}H_{71}O_{10}P$	778.4785	M+K	817.4416	-2.6	Lipidmaps
PG(O-18:0/14:0)	$C_{38}H_{77}O_9P$	708.5305	M+Ag109	817.4347	5.8	Lipidmaps
PG(O-20:0/12:0)	$C_{38}H_{77}O_9P$	708.5305	M+Ag109	817.4347	5.8	Lipidmaps
PG(O-16:0/16:0)	$C_{38}H_{77}O_9P$	708.5305	M+Ag109	817.4347	5.8	Lipidmaps

MS search for 837.6529 *m/z*

Compound	Formula	Compound MW	Adduct type	Adduct *m/z* calc	Delta [ppm]	Database
3-O-(6'-O-octadecanoyl-beta-D-glucopyranosyl)-cholest-5-en-3beta-ol	$C_{51}H_{90}O_7$	814.6687	M+Na	837.6579	-5.9	Lipidmaps
3-O-(6'-O-hexadecanoyl-beta-D-glucopyranosyl)-stigmast-5-en-3beta-ol	$C_{51}H_{90}O_7$	814.6687	M+Na	837.6579	-5.9	Lipidmaps
Stigmast-5-en-3beta-ol 3-0-(6'-O-hexadexcanoyl-beta-D-glucopyranoside)	$C_{51}H_{90}O_7$	814.6687	M+Na	837.6579	-5.9	Lipidmaps

MS search for 839.6733 *m/z*

Compound	Formula	Compound MW	Adduct type	Adduct *m/z* calc	Delta [ppm]	Database
3-O-(6'-O-(11Z,14Z-eicosadienoyl)-beta-D-glucopyranosyl)-cholest-5-en-3beta-ol	$C_{53}H_{90}O_7$	838.6687	M+H	839.6759	-3.1	Lipidmaps
3-O-(6'-O-(9Z,12Z-octadecadienoyl)-beta-D-glucopyranosyl)-stigmast-5-en-3beta-ol	$C_{53}H_{90}O_7$	838.6687	M+H	839.6759	-3.1	Lipidmaps
3-O-(6'-O-(9Z-octadecenoyl)-beta-D-glucopyranosyl)-stigmast-5,22E-dien-3beta-ol	$C_{53}H_{90}O_7$	838.6687	M+H	839.6759	-3.1	Lipidmaps

MS search for 1088.4206 *m/z*

Compound	Formula	Compound MW	Adduct type	Adduct *m/z* calc	Delta [ppm]	Database
CoA(22:1(13Z))	$C_{43}H_{76}N_7O_{17}P_3S$	1087.4231	M+H	1088.4304	-9	Lipidmaps

MS search for 1571.8431 *m/z*

Compound	Formula	Compound MW	Adduct type	Adduct *m/z* calc	Delta [ppm]	Database
KDNalpha2-3Galbeta1-3GalNAcbeta1-4Galbeta1-4Glcbeta-Cer(d18:1/20:0)	$C_{73}H_{132}N_2O_{31}$	1532.8814	M+K	1571.8446	-0.9	Lipidmaps
Galbeta1-3GalNAcbeta1-4(KDNalpha2-3)Galbeta1-4Glcbeta-Cer(d18:1/20:0)	$C_{73}H_{132}N_2O_{31}$	1532.8814	M+K	1571.8446	-0.9	Lipidmaps
KDNalpha2-3Galbeta1-3GlcNAcbeta1-3Galbeta1-4Glcbeta-Cer(d18:1/20:0)	$C_{73}H_{132}N_2O_{31}$	1532.8814	M+K	1571.8446	-0.9	Lipidmaps
KDNalpha2-6Galbeta1-3GlcNAcbeta1-3Galbeta1-4Glcbeta-Cer(d18:1/20:0)	$C_{73}H_{132}N_2O_{31}$	1532.8814	M+K	1571.8446	-0.9	Lipidmaps
KDNalpha2-3Galbeta1-4GlcNAcbeta1-3Galbeta1-4Glcbeta-Cer(d18:1/20:0)	$C_{73}H_{132}N_2O_{31}$	1532.8814	M+K	1571.8446	-0.9	Lipidmaps
KDNalpha2-6Galbeta1-4GlcNAcbeta1-3Galbeta1-4Glcbeta-Cer(d18:1/20:0)	$C_{73}H_{132}N_2O_{31}$	1532.8814	M+K	1571.8446	-0.9	Lipidmaps

MS search for 1966.979 *m/z*

Compound	Formula	Compound MW	Adduct type	Adduct *m/z* calc	Delta [ppm]	Database
Galbeta1-4(Fucalpha1-3)GlcNAcbeta1-6(Galbeta1-3)GalNAcbeta1-3Galalpha1-4Galbeta1-4Glcbeta-Cer(d18:1/18:0)	$C_{88}H_{157}N_3O_{42}$	1928.0242	M+K	1966.9873	-4.2	Lipidmaps
Galalpha1-3(Fucalpha1-2)Galbeta1-4GlcNAcbeta1-3Galbeta1-4GlcNAcbeta1-3Galbeta1-4Glcbeta-Cer(d18:1/18:0)	$C_{88}H_{157}N_3O_{42}$	1928.0242	M+K	1966.9873	-4.2	Lipidmaps

Delta - calculated as $[(m/z_{calc.} - m/z_{meas.})*10^6]/m/z_{calc.}$, where m/z_{calc} calculated compound *m/z*; m/z_{meas} measured *m/z*.
